# EML4-ALK induces cellular senescence in mortal normal human cells and promotes anchorage-independent growth in hTERT-transduced normal human cells

**DOI:** 10.1186/s12885-021-07905-6

**Published:** 2021-03-24

**Authors:** Akihiko Miyanaga, Masaru Matsumoto, Jessica A. Beck, Izumi Horikawa, Takahiro Oike, Hirokazu Okayama, Hiromi Tanaka, Sandra S. Burkett, Ana I. Robles, Mohammed Khan, Delphine Lissa, Masahiro Seike, Akihiko Gemma, Hiroyuki Mano, Curtis C. Harris

**Affiliations:** 1 Laboratory of Human Carcinogenesis, Center for Cancer Research, National Cancer Institute, National Institutes of Health, 37 Convent Drive, Room 3068A, Bethesda, MD 20892 USA; 2 Department of Pulmonary Medicine and Oncology, Graduate School of Medicine, Nippon Medical School, Tokyo, Japan; 3 Medical and Molecular Genetics, Indiana University School of Medicine, Indianapolis, Indiana USA; 4 Molecular Cytogenetic Core Facility, Mouse Cancer Genetics Program, Center for Cancer Research, National Cancer Institute, National Institutes of Health, Frederick, MD USA; 5 Division of Cellular Signaling, National Cancer Center Research Institute, Tokyo, Japan

**Keywords:** EML4-ALK, Lung cancer, Senescence, Anchorage-independent growth, hTERT, DNA damage

## Abstract

**Background:**

Chromosomal inversions involving anaplastic lymphoma kinase (*ALK*) and echinoderm microtubule associated protein like 4 (*EML4*) generate a fusion protein EML4-ALK in non-small cell lung cancer (NSCLC). The understanding of EML4-ALK function can be improved by a functional study using normal human cells.

**Methods:**

Here we for the first time conduct such study to examine the effects of EML4-ALK on cell proliferation, cellular senescence, DNA damage, gene expression profiles and transformed phenotypes.

**Results:**

The lentiviral expression of EML4-ALK in mortal, normal human fibroblasts caused, through its constitutive ALK kinase activity, an early induction of cellular senescence with accumulated DNA damage, upregulation of p16^INK4A^ and p21^WAF1^, and senescence-associated β-galactosidase (SA-β-gal) activity. In contrast, when EML4-ALK was expressed in normal human fibroblasts transduced with telomerase reverse transcriptase (hTERT), which is activated in the vast majority of NSCLC, the cells showed accelerated proliferation and acquired anchorage-independent growth ability in soft-agar medium, without accumulated DNA damage, chromosome aberration, nor *p53* mutation. EML4-ALK induced the phosphorylation of STAT3 in both mortal and hTERT-transduced cells, but RNA sequencing analysis suggested that the different signaling pathways contributed to the different phenotypic outcomes in these cells. While EML4-ALK also induced anchorage-independent growth in hTERT-immortalized human bronchial epithelial cells in vitro, the expression of EML4-ALK alone did not cause detectable in vivo tumorigenicity in immunodeficient mice.

**Conclusions:**

Our data indicate that the expression of hTERT is critical for EML4-ALK to manifest its in vitro transforming activity in human cells. This study provides the isogenic pairs of human cells with and without EML4-ALK expression.

**Supplementary Information:**

The online version contains supplementary material available at 10.1186/s12885-021-07905-6.

## Background

The *ALK* gene undergoes chromosomal translocations and fusions with other genes to generate oncogenic fusion proteins in non-small cell lung carcinoma (NSCLC) and other malignancies [1–3]. The *EML4* gene is the most frequent fusion partner of *ALK* in NSCLC, resulting in an oncogenic fusion protein EML4-ALK [3, 4]. The tumors with EML4-ALK rarely have the other genetic changes such as mutations of *EGFR*, *KRAS* and *TP53* [5], suggesting that their development strongly depends on the oncogenic fusion protein. The inhibition of the ALK tyrosine kinase activity has led to major advances in treatment of patients with ALK fusion-positive tumors [2, 6, 7].

The basic understanding of the biological activities of ALK fusion proteins remains incomplete, partly due to the lack of functional studies using normal human cells. Previous studies used murine cell lines, human cancer-derived cell lines or chromosomally abnormal immortalized human cells [4, 8–10]. This study for the first time investigates the function of EML4-ALK in mortal, normal human cells, as well as in hTERT-transduced normal human cells. Our data show the different activities of EML4-ALK in mortal and hTERT-transduced normal human cells and provide mechanistic insight into its role during human carcinogenesis.

## Methods

### Cells and cell culture

CRL-2097, BJ and MRC-5 and NIH/3 T3 were obtained from ATCC (Manassas, VA, USA) and maintained in DMEM supplemented with 10% FBS. HBET1 [11] and their derived cells were maintained in LHC-9 medium (Thermo Fisher Scientific, Waltham, MA USA) supplemented with 2 mM L-glutamine. H3122 was obtained from the NCI Repository of Tumor Cell Lines (Frederick, MD, USA) and maintained in RPMI 1640 medium supplemented with 10% FBS. For tetracycline (Tet)-inducible gene expression, doxycycline (Dox, at 1 μg/ml) was added. In continuous culture of human fibroblasts to examine cellular replicative lifespan, the cells were passaged at a split ratio of 1:4 (or 1:2 at later passages when approaching senescence). The number of population doubling levels (PDL) achieved between passages was determined by log_2_ (“number of cells obtained” divided by “number of cells inoculated”) [11] and data were presented as means ± s.d. from biological triplicates. Crizotinib was purchased from Selleck Chemicals (Houston, TX, USA) and used at a concentration of 25 nM. For all cells, culture medium was changed every 48 h. For cells treated with Dox or Dox plus Crizotinib, they were continuously included in the medium.

SA-β-gal staining was performed using the kit purchased from Cell Signaling Technology (Danvers, MA, USA).

### Lentiviral and retroviral expression vectors and vector transduction

pLenti3.3/TR and a lentiviral expression vector pLenti6.3/TO/V5-DEST were from Thermo Fisher Scientific. The EML4-ALK cDNA variant 1 [3, 4] was transferred into pLenti6.3/TO/V5-DEST for its inducible (with pLenti3.3/TR) and constitutive expression (without pLenti3.3/TR). The red fluorescent protein (RFP) cDNA was also transferred from pLOC (Open Biosystem, Lafayette, CO, USA) to pLenti6.3/TO/V5-DEST, generating the control vector. EML4-ALK (K589M) in pLenti6.3/TO/V5-DEST was generated via site-directed mutagenesis using the QuikChange II XL kit (Stratagene, Carlsbad, CA, USA). The retroviral expression vector for H-RasV12 (in pBabe vector) was a gift from Dr. Manuel Serrano (IRB Barcelona, Spain) [12]. The retroviral vector for hTERT (pCLXSN-hTERT) was previously described [11] and used to generate hTERT-immortalized BJ (hTERT-BJ) and HBET1 previously [11, 13] and hTERT-transduced CRL-2097 (hTERT-CRL-2097) in this study. The preparation of vector supernatants and the vector transduction were performed as previously described [11, 14, 15]. Two days after transduction, the cells were selected with puromycin (for pBabe, 1 μg/ml; Sigma-Aldrich), G418 (for pLenti3.3/TR and pCLXSN-hTERT, 500 μg/ml; Sigma-Aldrich) or blasticidin (for pLenti6.3/TO/V5-DEST, 2 μg/ml; Thermo Fisher Scientific).

The coding sequences in all newly constructed vectors were fully sequenced for confirmation.

### Protein lysates and western blot analysis

Cells were lysed in 20 mM Tris-HCl (pH 7.5) / 150 mM NaCl / 0.1% SDS / 1% NP-40 / 1 mM EDTA containing the Complete protease inhibitor cocktail (Roche Diagnostics, Indianapolis, IN, USA). Western blot analysis was performed as described previously [11, 16]. Signal detection was performed using the SuperSignal West Dura chemiluminescence substrate (Thermo Fisher Scientific) and the images were captured using the ChemiDoc Imager (Bio-Rad, Hercules, CA, USA). Quantitative image analysis used the ImageJ software (http://rsb.info.nih.gov/ij/). Primary antibodies used were as follows: anti-ALK (#3633, Cell Signaling Technology; 1:2000); anti-phosho-ALK (#3341, Cell Signaling Technology; 1:1000); anti-STAT3 (#9139, Cell Signaling Technology; 1:1000); anti-phospho-STAT3 (#9145, Cell Signaling Technology; 1:2000); anti-AKT (#9272, Cell Signaling Technology; 1:1000); anti-phospho-AKT (#9271, Cell Signaling Technology; 1:1000); anti-Src (#2123, Cell Signaling Technology; 1:1000); anti-phospho-Src (#6943, Cell Signaling Technology; 1:1000); anti-Erk1/2 (#9102, Cell Signaling Technology; 1:1000); anti-phosho-Erk1/2 (#9101, Cell Signaling Technology; 1:1000); anti-c-H-Ras (#OP23, Calbiochem; 1:30); anti-p16^INK4A^ (sc-468, Santa Cruz Biotechnology, Dallas, TX, USA; 1:1000); anti-p21^WAF1^ (#2947, Cell Signaling Technology; 1:1000); anti-p53 (DO-1, sc-126, Santa Cruz Biotechnology, Dallas, TX, USA; 1:1000); anti-phospho-p53 (Ser15) (#9284, Cell Signaling Technology; 1:1000); and anti-GAPDH (sc-166,574, Santa Cruz Biotechnology; 1:1000.

### Immunofluorescence staining

Cells (3.0 × 10^4^ cells/well) were washed with PBS and fixed for 10 min with 4% paraformaldehyde, followed by permeabilization with 0.25% Triton-X-100 for 10 min. Incubation with anti-γ-H2AX antibody (Catalog #05–636, Sigma Aldrich; 1:1000) and then with an Alexa Fluor 488-conjugated secondary antibody (Catalog #A-21202; Thermo Fisher Scientific; 1:400), washing procedures, and mounting with Antifade with DAPI (Vectashield, Burlingame, California) were as previously described [17, 18]. Digital images were acquired and analyzed using confocal microscopy (Zeiss 780) and ZEN software. The total signal intensity of γ-H2AX immunofluorescence per microscopic field was determined in unmerged γ-H2AX images using Image J software and expressed as the mean signal intensity per DAPI+ nucleus manually quantified in five high powered fields (40x).

### Anchorage-independent colony formation in soft-agar medium

Cells (1000 cells/well) were seeded on 12-well plates in 1 ml of medium containing 0.35% agarose (SeaPlaque low-melting-temperature agarose, Lonza Biosciences, Alpharetta, GA, USA) over 1-ml volume of base layer consisting of the same culture medium and 0.5% agarose. Colonies of approximately 50 μm in diameter or larger were counted on day 21.

### Telomere length measurement by quantitative PCR (qPCR)

Telomere length was measured by the monochrome multiplex qPCR (MMQPCR) method as described previously [19]. Relative telomere length was express as a ratio of the quantity of telomeric DNA (T) normalized to the quantity of multiple copy sequence DNA (mcs), yielding T/M ratio. Intra- and inter-assay coefficient of variations (CVs) were 4.1 and 10.7%, respectively.

### G-band karyotyping and spectral karyotyping (SKY)

Treatment with Colcemid (10 μg/ml, KaryoMax, Invitrogen, Carlsbad, CA, USA), hypotonic treatment (0.075 M KCl), fixation with methanol/acetic acid (3,1), slide preparation and G-banding were performed as previously described [20] and the images were captured and analyzed with the HiBand system (Applied Spectral Imaging, Carlsbad, CA, USA). For spectral karyotyping (SKY), the slides were processed using the 24-color Human SKY Paint kit (Applied Spectral Imaging) according to the manufacturer’s protocol. Spectral images of the hybridized metaphases were acquired using the HyperSpectral Imaging system (Applied Spectral Imaging) and analyzed using the HiSKY v.7.2 acquisition software (Applied Spectral Imaging). All cell lines were analyzed by G-band karyotyping. hTERT-CRL-2097, hTERT-CRL-2097 + EML4-ALK, its derived soft-agar clones #1 and #2, and HBET1 were also analyzed by SKY.

### Tumorigenicity in vivo

Female NOD.SCID/Ncr mice were obtained from Charles River Laboratories (#560; Germantown, MD, USA), and were maintained under specific pathogen-free conditions and the animals had free access to feedstuff and water under 12:12 light/dark cycles, 22 and 30–70% humidity. These mice at 6 to 10 weeks of age were subcutaneously injected with cells (5 × 10^6^ per flank) in 50% Matrigel (no. 354248, Corning, NY, USA) at both flanks: one side with EML4-ALK-expressing cells or their derived soft-agar clones; and the other side with vector control cells. For injection of these cells, mice were anesthetized using isoflurane in accordance with National Cancer Institute (NCI) Animal Care and Use Committee (ACUC) guidelines. As a positive control, NIH/3 T3 cells expressing EML4-ALK were injected at both flanks of two mice (2 × 10^6^ per flank). Tumor size and body weight were measured twice a week until 8 weeks or tumor size excess 20 mm. To ameliorate the suffering of mice observed throughout experimental studies, mice were euthanized by CO_2_ inhalation. After 8 weeks of administration, the mice were sacrificed and photographed, and the tumors were removed and weighed. This experiment was approved by the *NCI* ACUC (LHC-012-D).

### RNA isolation, reverse transcription (RT)-PCR and sanger sequencing of the entire coding region of p53 mRNA

Total RNA isolation, reverse transcription, and 1st strand cDNA synthesis with random hexamers were carried out as previously described [11, 15]. PCR amplification of the entire coding region of p53 mRNA was carried out using the Platinum *Taq* DNA Polymerase High Fidelity (Thermo Fisher Scientific) with the primers 5′-ATG GAG GAG CCG CAG TCA-3′ and 5′-GTC AGT CTG AGT CAG GCC CTT C-3′. The amplified PCR product was sequenced using the BigDye Terminator v1.1 Cycle Sequencing kit (Thermo Fisher Scientific) with 5′-AGT ACG TGC AAG TCA CAG-3′, 5′-CGT CCC AAG CAA TGG ATG-3′ and 5′-CTC ACC ATC ATC ACA CTG G-3′.

### RNA sequencing (RNA-seq) analysis

RNA samples were treated with DNase I (no. 18068015, Thermo Fisher Scientific), followed by purification with RNeasy MinElute Cleanup kit (Qiagen). The RNA integrity numbers (RIN) of the samples on the *Agilent 2100 Bioanalyzer (Agilent* Technologies*)* were 9.3 to 10.0. All the following steps of the RNA-seq experiment were carried out at the CCR Sequencing Facility (Leidos Biomedical Research, Frederick, MD, USA). Sequencing libraries were prepared using the TruSeq Stranded mRNA Library Prep kit (Illumina, San Diego, CA, USA) and were sequenced with paired-end reads of 150 bp on HiSeq3000/4000 sequencer (Illumina) to obtain at least 30 million read pairs per sample. Reads were trimmed for adaptors and low-quality bases using Trimmomatic [21] and were aligned with the reference genome (Human-hg38) and the annotated transcripts (Gencode_v24) using STAR (https://github.com/alexdobin/STAR). The gene expression quantification analysis was performed using STAR/RSEM tools (http://deweylab.github.io/RSEM/) to obtain raw read counts and normalized read counts (RPKM) for each gene.

Differentially expressed genes were identified using DESeq2 in R [22] with a false discovery rate (*FDR*) cutoff of *0.0*1, and analyzed by gene enrichment analysis for KEGG (https://www.genome.jp/kegg/) and Reactome Pathways (https://reactome.org/) using DAVID v6.8 (https://david.ncifcrf.gov/). Enriched pathways were identified according to FDR ≤ 0.05.

### MicroRNA quantification by qPCR

PCR was performed by TaqMan MicroRNA Assays as manufacturer’s instructions. Primers used were as follows: miR-21 (Taqman microRNA assay, hsa-miR-21, PN 4440887); RNU48 (Taqman microRNA assay, RNU48, PN 4440887).

### Data mining of publicly available lung adenocarcinoma datasets

For the TCGA dataset [23], mRNA expression from RNA Seq V2 RSEM and tumor characteristics were downloaded from cBioPortal for 510 patients with lung adenocarcinoma. Log2-transformed RSEM values for hTERT were used for the analyses.

For the microarray dataset GSE31210 [24], data and sample characteristics were downloaded from the Gene Expression Omnibus for 226 patients with lung adenocarcinoma using the R package GEOquery. The raw intensity values were processed and normalized with Robust Multi-Array Average (RMA) using the R package oligo. Affymetrix IDs were mapped to HUGO Gene Nomenclature Committee IDs. Resulting RMA expression values for hTERT were used for the analyses.

### Quantitative RT-PCR analysis

Quantitative RT-PCR analysis to confirm the RNA-seq data was performed by using TaqMan Assay as manufacture’s instruction. Primer used was as follows: IL1B (Taqman Hs01555410_m1, Cat 4,331,182), CXCL8 (Taqman Hs00174103_m1, Cat 4,331,182), GAPDH (Taqman Hs02786624_g1, Cat 4,331,182), PLAU (Taqman Hs01547050_m1, Cat 4,331,182), PLAT (Taqman Hs 00938315_m1, Cat 4,331,182) and A2M (Taqman Hs 00929971_m1, Cat 4,331,182).

### Statistical analysis

Data are presented as means ± s.d. from at least three biological replicates. Statistical significance was evaluated using unpaired 2-tailed Student’s *t*-test. * *P* < 0.05, ** *P* < 0.01 and *** *P* < 0.001.

## Results

### EML4-ALK causes the early induction of cellular senescence in normal, mortal human fibroblasts

**Fig. 1:**
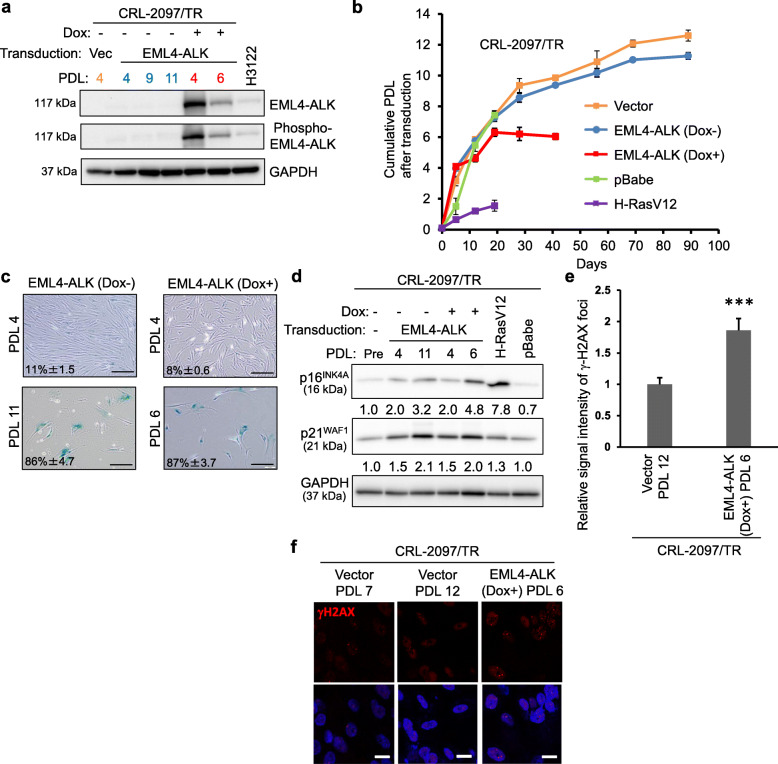
EML4-ALK induces early onset of cellular senescence in mortal normal human fibroblasts. CRL-2097/TR were transduced with an inducible lentiviral vector of EML4-ALK or its control vector (Vec). Shown PDL is after transduction. **a** Western blot analysis confirming the inducible expression of EML4-ALK. The EML4-ALK- or Vec-transduced cells with (+) or without (−) doxycycline induction (Dox) were examined at indicated PDL for protein expression levels of total EML4-ALK and phosphorylated EML4-ALK. GAPDH (glyceraldehyde 3-phosphate dehydrogenase) was a loading control. H3122 (EML4-ALK expressing cells). Full-length blots are presented in Supplementary Figure. **b** Replicative lifespan of transduced CRL-2097/TR fibroblasts. Cumulative PDL after transduction were plotted to days after transduction. According to ATCC’s PDL counting, CRL-2097/TR at the initiation of this experiment corresponded to PDL 43 in total. The proliferation arrest of Vector or Dox (−) control cells at PDL 11 or 12 after transduction, corresponding to PDL 54 or 55 in total, is consistent with the information from ATCC that this strain senesces at PDL 56. **c** SA-β-gal staining of CRL-2097/TR fibroblasts with (Dox+) and without (Dox-) induced expression of EML4-ALK. Representative images in proliferative phase (both at PDL 4) and growth arrested phase (Dox + at PDL 6 and Dox- at PDL 11) are shown. Percentages of SA-β-gal positive cells (means ± s.d.) were from biological triplicate, in each of which more than 300 cells were observed. Scale bars, 20 μm. **d** Western blot analysis of p16^INK4A^ and p21^WAF1^. The EML4-ALK-transduced CRL-2097/TR fibroblasts with (+) and without (−) Dox induction at indicated PDL, in parallel to the cells before transduction (Pre), and those retrovirally transduced with H-RasV12 and its control vector pBabe (Supplementary Fig. S1A-B) were examined. Quantitative expression levels of p16^INK4A^ and p21^WAF1^ (normalized with GAPDH) are shown in relative to the levels in the cells before transduction (Pre). **e** Early and accelerated accumulation of DNA damage in cells expressing EML4-ALK. Senescent CRL-2097/TR with control vector (PDL 12) and those with Dox-induced EML4-ALK expression (PDL 6) were examined for γ-H2AX foci. The data (signal intensity per cell) are mean ± s.d. from triplicates and shown relative to the vector control. **f** Representative images of γ-H2AX immunofluorescence staining in the cells shown in (**e**). The lower panels were merged with DAPI. CRL-2097/TR with control vector (PDL 7) is also shown as comparison. Note that the level of γ-H2AX foci in natural replicative senescence (Vector, PDL 12) is consistent with that previously reported [25]. Scale bars, 20 μm

We transduced normal human fibroblasts CRL-2097, which are mortal, or have a limited replicative lifespan, with a lentiviral vector expressing the Tet repressor (generating CRL-2097/TR), and then with either a lentiviral vector encoding EML4-ALK or the control vector (Fig. 1a). While the control cells with no EML4-ALK expression reached approximately 11 or 12 population doubling levels (PDL) before proliferation arrest, the EML4-ALK-expressing cells ceased to proliferate earlier at PDL 6 after lentiviral transduction (Fig. 1b). Both the control and EML4-ALK-expressing cells, upon proliferation arrest, were similarly positive for SA-β-gal (Fig. 1c). In addition to the cells shown in Fig. 1a (observed through day 88 or 40 in Fig. 1b), those retrovirally transduced with oncogenic Ras (H-RasV12) and its control vector (pBabe) were examined (Supplementary Fig. S1A; observed through day 20 in Fig. 1b) and H-RasV12-induced senescence was observed (Supplementary Fig. S1B). Thus, the early induction of cellular senescence by EML4-ALK was reminiscent of oncogenic Ras-induced senescence [12], although H-RasV12 induced-senescence occurred earlier in this CRL-2097 strain (Fig. 1b, Supplementary Fig. S1B). The high level of EML4-ALK expression might have been selected against during entry into senescence (Dox + at PDL 6 in Fig. 1a; also see below Discussion).

### p16^INK4A^ and p21^WAF1^ are upregulated in both natural replicative senescence and EML4-ALK-induced early senescence

The expression levels of p16^INK4A^ and p21^WAF1^, two markers of cellular senescence [26, 27], were examined in CRL-2097/TR with EML4-ALK or H-RasV12 expression, along with their control cells (Fig. 1d). The p16^INK4A^ protein expression was increased during the replicative lifespan in both EML4-ALK-expressing and control cells, although its levels at senescence were lower than observed in H-RasV12-induced senescence. An increase in p21^WAF1^ protein expression was also observed similarly in the EML4-ALK-expressing and control cells, and to a lesser degree in H-RasV12-induced senescence. These findings suggest that the similar levels of upregulation of p16^INK4A^ and p21^WAF1^ occur with the EML4-ALK-induced senescence and natural replicative senescence, although the former undergoes fewer PDL than the latter to achieve those expression levels.

### The EML4-ALK-induced senescence is associated with accumulated DNA damage

A phosphorylated H2AX (γ-H2AX) [13] was detected by immunofluorescence staining in CRL-2097/TR fibroblasts with EML4-ALK expression (EML4-ALK-Dox + in Fig. 1b) and with the control vector (Vector in Fig. 1b) when approaching senescent proliferation arrest (at PDL 6 and PDL 12, respectively) (Fig. 1e-f). The early induction of cellular senescence in the EML4-ALK-expressing cells was associated with significantly more accumulation of γ-H2AX foci (Fig. 1e-f), suggesting that EML4-ALK-induced senescence accompanies an accelerated rate of persistent DNA damage per PDL compared with natural replicative senescence. The level of p53 protein phosphorylated at serine 15, a target residue of the ATM kinase signaling from DNA damage to cellular senescence [28–30], was increased in both control and EML4-ALK-expressing cells upon senescence (Supplementary Fig. S2A).

A quantitative PCR-based measurement of telomere length showed that the vector control cells underwent progressive telomere shortening through their replicative senescence at PDL 12 (Supplementary Fig. S2B). The EML4-ALK-expressing cells had the telomere length shorter than that of the original cells before lentiviral transduction, similar to that of the control cells at a comparable PDL (PDL 7) and longer than that of the replicatively senescent control cells at PDL 12 (Supplementary Fig. S2B). These data suggest that normal, mortal human fibroblasts with and without EML4-ALK expression undergo telomere shortening at similar rates per PDL, and that the accelerated accumulation of DNA damage in the EML4-ALK-expressing cells may be of non-telomeric origin [13].

### The EML4-ALK-induced senescence depends on its ALK kinase activity

**Fig. 2:**
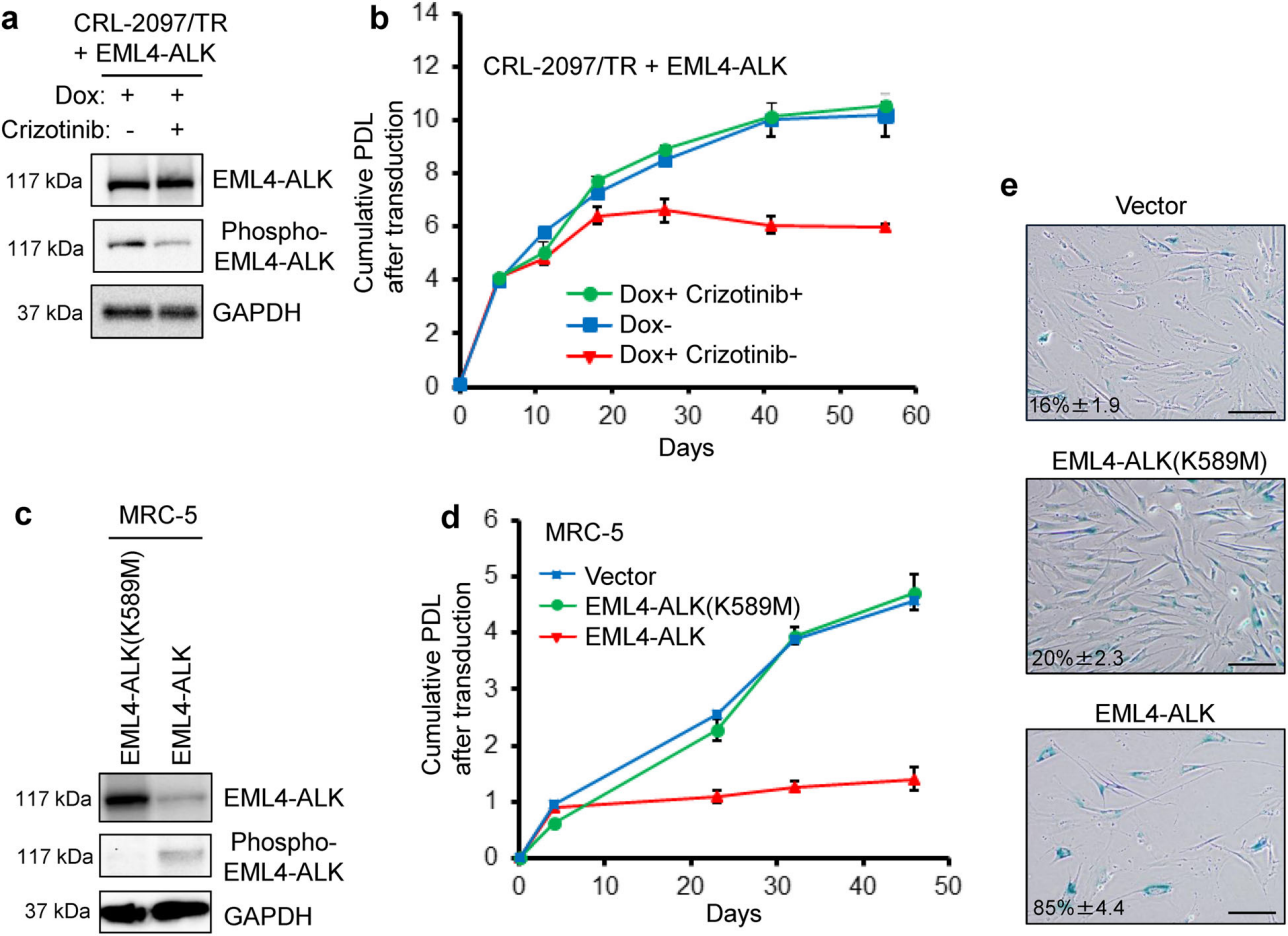
The kinase activity of EML4-ALK is required for early induction of cellular senescence. **a** Western blot analysis showing a decrease in EML4-ALK autophosphorylation by an ALK TKI Crizotinib. CRL-2097/TR fibroblasts with Dox-induced expression of EML4-ALK were maintained in culture in the absence (−) or presence (+) of 25 nM Crizotinib and examined for levels of total EML4-ALK (top) and phosphorylated EML4-ALK (middle). Dox was added at day 0 and Crizotinib was added at day 5 and both remained included throughout the experiment with medium change every 48 h. The cells were harvested at day 40 (at PDL 6 or 10) for western blot. **b** Abrogation of early senescence by Crizotinib. The Dox-treated cells (Dox+) with and without Crizotinib (+ and -) shown in (**a**), along with the untreated cells (Dox-), were examined for cumulative PDL after transduction as in Fig. 1b. **c** Western blot analysis of MRC-5 fibroblasts that express wild-type EML4-ALK and the kinase-dead mutant (K589M). MRC-5, a second strain of mortal normal human fibroblasts, were transduced with the lentiviral vector of EML4-ALK (the same vector as used above, which drives constitutive expression in the absence of a Tet repressor) and its K589M mutant derivative. Western blot analysis was performed as in (**a**). **d** Dependence of early senescence on the kinase activity of EML4-ALK. The transduced MRC-5 fibroblasts shown in (**c**), along with the vector control-transduced cells, were examined for cumulative PDL after transduction, as in (**b**) and Fig. 1b. Note that this experiment used late-passage MRC-5 with fewer PDL remaining until natural replicative senescence, compared with CRL-2097/TR used in the above experiments. **e** Representative images of SA-β-gal staining with quantitative data of positive cells (mean ± s.d., as in Fig. 1c). The cells shown in (**d**) were examined at day 22. Scale bars, 20 μm

The EML4-ALK-expressing CRL-2097/TR with Dox addition were treated with an ALK tyrosine kinase inhibitor (TKI) Crizotinib at 25 nM (a concentration close to the reported IC_50_ value [31]) and were monitored for cell proliferation. The treatment with Crizotinib inhibited the autophosphorylation of EML4-ALK while not affecting the expression level of EML4-ALK, confirming its TKI activity against the ALK kinase activity (Fig. 2a). The expression of EML4-ALK without Crizotinib reproducibly induced the proliferation arrest at PDL 6 (Fig. 2b). In contrast, the Crizotinib-treated cells bypassed this early proliferation arrest and underwent approximately 4 more PDL, similarly to the control cells without EML4-ALK expression (Fig. 2b).

Another normal human fibroblast strain MRC-5 (without a Tet repressor) was transduced with the lentiviral vector encoding wild-type EML4-ALK or a kinase-dead mutant of EML4-ALK (K589M) [4], along with the control vector. The K589M mutant of EML4-ALK was constitutively expressed at a higher level than the wild-type counterpart but was not autophosphorylated (Fig. 2c). Since the MRC-5 fibroblasts used in this experiment were closer to natural replicative senescence than the CRL-2097 fibroblasts used above, the control vector-transduced cells underwent at most 5 PDL before they ceased to proliferate (Fig. 2d). The expression of wild-type EML4-ALK, again in these fibroblasts approaching natural replicative senescence, caused earlier induction of proliferation arrest (Fig. 2d) with SA-β-gal (Fig. 2e), which was similar to oncogenic Ras-induced senescence (Supplementary Fig. S1C-D). Importantly, the K589M mutant-expressing cells did not show early proliferation arrest and behaved like the control vector-transduced cells (Fig. 2d-e), further supporting that the ALK kinase activity mediates the early induction of cellular senescence by EML4-ALK.

### EML4-ALK promotes anchorage-independent growth in hTERT-transduced normal human fibroblasts

**Fig. 3:**
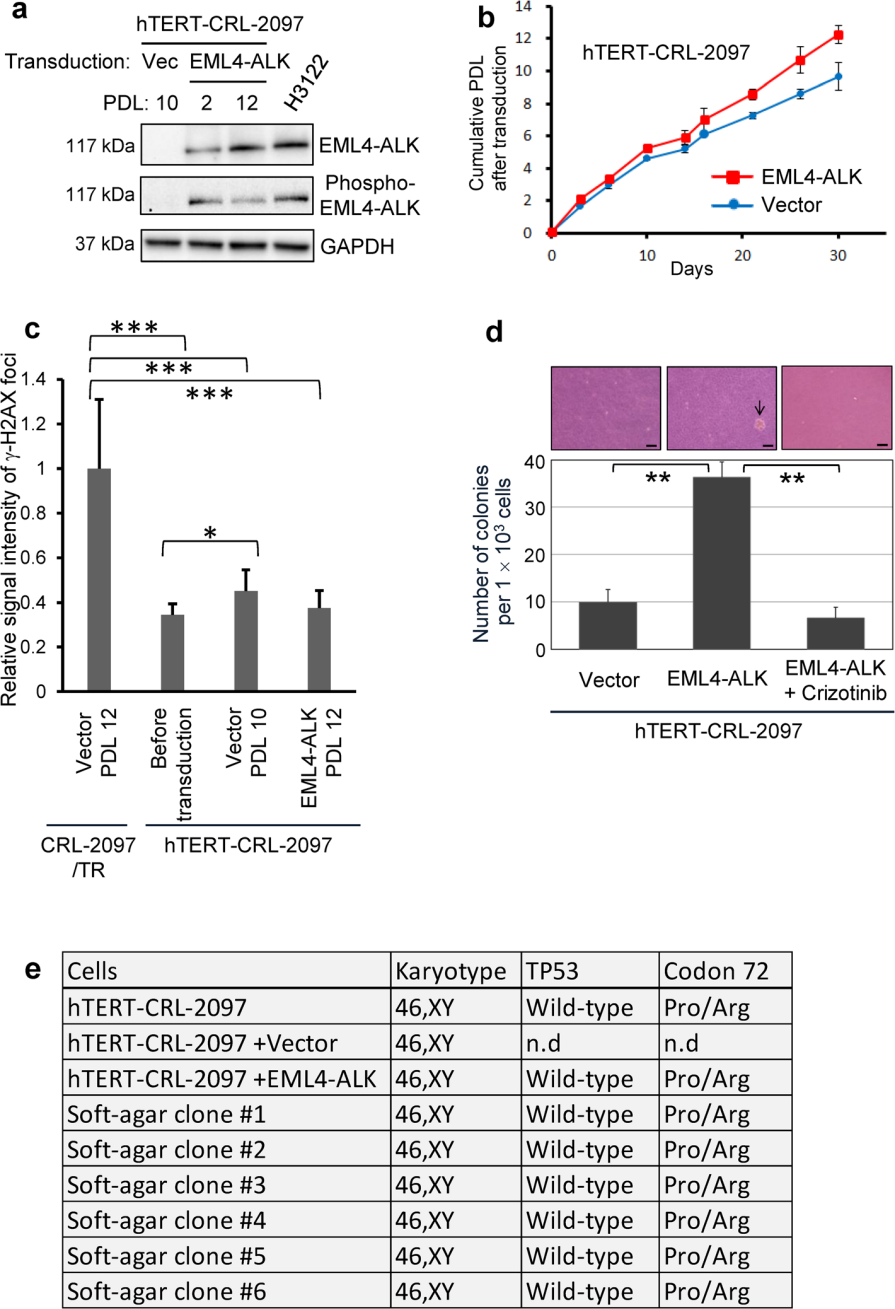
EML4-ALK promotes anchorage-independent growth in hTERT-transduced normal human fibroblasts. **a** hTERT-CRL-2097 were transduced with the EML4-ALK lentiviral vector (for the constitutive expression in the absence of a Tet repressor) or the control vector (Vec). Western blot analysis was performed in the cells at indicated PDL after transduction, as in Fig. 1a, with H3122 as a positive control. **b** Cell proliferation curves of hTERT-CRL-2097 with the expression of EML4-ALK or the control vector. Cumulative PDL were calculated and plotted to days after transduction, as above. **c** No accumulation of DNA damage by EML4-ALK in hTERT-transduced fibroblasts. The hTERT-CRL-2097 cells expressing EML4-ALK (PDL 12), with the control vector (PDL 10) and before transduction were examined for γ-H2AX foci as in Fig. 1e. The CRL-2097/TR fibroblasts at senescence (Vector at PDL 12 in Fig. 1b and e) were again examined for comparison and shown in parallel. The data quantification and analysis were as in Fig. 1e. * *P* < 0.05; *** *P* < 0.001. **d** Anchorage-independent growth of hTERT-CRL-2097 cells expressing EML4-ALK, with and without 25 nM Crizotinib. Those cells with the control vector were plated in soft-agar medium and examined for colony formation at day 21. Numbers of colonies per 1 × 10^3^ cells plated (means ± s.d. from biological triplicate) are shown with representative images without or with a colony (arrow). Scale bars, 50 μm. ** *P* < 0.01. **e** Summary of karyotype and the status of the *TP53* gene in EML4-ALK-expressing hTERT-CRL-2097 fibroblasts and their derived cell clones isolated and established from soft-agar colonies (#1 to #6). n.d., not determined

Although EML4-ALK-positive NSCLC rarely have other accompanying genetic alterations [5], they still have a mechanism to maintain telomere length and function, in most cases via hTERT activation [32]. Consistent with the previous findings [33], ALK fusion-positive NSCLC tumor tissues, as well as negative ones, were confirmed to express hTERT (Supplementary Fig. S3A and B). We thus hypothesized that EML4-ALK might cooperate with the expression of hTERT, leading to telomerase activation and cell immortalization, to cause cellular transformation in normal human cells. To test this hypothesis, hTERT was retrovirally transduced into CRL-2097 to establish an hTERT-transduced normal human cell line (hTERT-CRL-2097), which maintained elongated telomeres (Supplementary Fig. S4A). This cell line had normal karyotype (Supplementary Fig. S4B), maintained normal p16^INK4A^ response to oncogenic Ras (Supplementary Fig. S4C) and retained wild-type *TP53* (below in Fig. 3e), thus not coincident with the changes frequently associated with human cell immortalization. These hTERT-CRL-2097 cells were transduced with the wild-type EML4-ALK vector or the control vector, and the constitutive expression and autophosphorylation of the EML4-ALK protein was confirmed (Fig. 3a). Unlike in mortal CRL-2097, the expression of EML4-ALK in this hTERT-transduced cell line did not induce senescent proliferation arrest, but instead resulted in accelerated cell proliferation (Fig. 3b). The hTERT-CRL-2097 cells, with or without EML4-ALK expression, accumulated no or little DNA damage (γ-H2AX foci in Fig. 3c and Supplementary Fig. S5), consistent with no induction of cellular senescence and in contrast to DNA damage accumulation in mortal CRL-2097 fibroblasts (the leftmost bar in Fig. 3c; and Fig. 1e above). Furthermore, the anchorage-independent formation of cell colonies in soft-agar medium was enhanced in the EML4-ALK-expressing hTERT-CRL-2097 cells, which was inhibited by treatment with the ALK TKI Crizotinib (Fig. 3d).

In another hTERT-transduced normal human fibroblast cell line, hTERT-BJ (Supplementary Fig. S6A), the expression of wild-type EML4-ALK, but not of the K589M mutant, accelerated cell proliferation (Supplementary Fig. S6B) and promoted anchorage-independent growth in soft agar (Supplementary Fig. S6C). The ability of wild-type EML4-ALK to promote anchorage-independent growth was again abrogated by treatment with the ALK TKI Crizotinib (Supplementary Fig. S6C). From our reproducible results in two lines of hTERT-transduced normal human fibroblasts, we conclude that EML4-ALK has in vitro transformation activity in these cells through its constitutive ALK kinase activity.

### The EML4-ALK-induced anchorage-independent growth occurs without chromosome aberrations and without loss or mutation of the *TP53* gene

We performed karyotype analysis in hTERT-CRL-2097 cells transduced with the control vector, those expressing EML4-ALK and six of their derived clones isolated from soft-agar culture. All of these cells examined, as well as the original hTERT-CRL-2097 cells (as mentioned above and Supplementary Fig. S4B), maintained normal male karyotype 46, XY (Fig. 3e and Supplementary Fig. S7A-B). All cell lines listed in Fig. 3e had normal male karyotype 46, XY by G-banding (at least 10 well-spread metaphases per line were examined; an example is shown in Supplementary Fig. S7A). hTERT-CRL-2097, hTERT-CRL-2097 + EML4-ALK and soft-agar clones #1 and #2 were also confirmed by SKY to have 46, XY (again, at least 10 metaphases per line were examined; an example is shown in Supplementary Fig. S7B). By direct sequencing of the RT-PCR products, the entire coding region of *TP53* was shown to be wild-type without a clonal homozygous or heterozygous mutation. The polymorphic codon 72 of *TP53* was heterozygous (Pro/Arg) in the original hTERT-CRL-2097 and remained heterozygous (Pro/Arg) in hTERT-CRL-2097 expressing EML4-ALK and their derived soft-agar clones (examples are shown in Supplementary Fig. S7C), indicating that no chromosome instability nor loss of heterozygosity (LOH) occurred at *TP53* during EML4-ALK-induced acquisition of anchorage-independent growth.

Although loss or mutation of the *TP53* gene is frequently associated with cell transformation and carcinogenesis [34], all of the EML4-ALK-expressing cells and their derived soft-agar clones had the wild-type *TP53* sequence without any homozygous or heterozygous mutation (Fig. 3e). The original hTERT-CRL-2097 cells showed Pro/Arg heterozygosity at the polymorphic codon 72, which was maintained in the EML4-ALK-expressing cells and all the soft-agar clones (Fig. 3e and Supplementary Fig. S7C), indicating that there was no loss of a *TP53* allele in these cells. The p16^INK4A^ pathway, which is frequently impaired during cell transformation and carcinogenesis [27, 35], was suggested to remain intact during the EML4-ALK-mediated cell transformation by the finding that oncogenic Ras-induced p16^INK4A^ upregulation [12] was observed in the EML4-ALK-expressing cells (Supplementary Fig. S7D).

### STAT3 is phosphorylated by EML4-ALK in both mortal and hTERT-transduced normal human fibroblasts

**Fig. 4:**
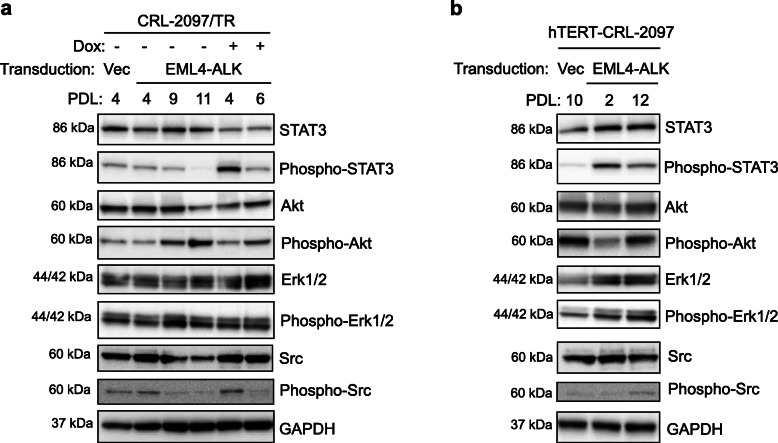
STAT3 is phosphorylated by EML4-ALK in both mortal and hTERT-transduced normal human fibroblasts. The same set of CRL-2097/TR-derived cells as in Fig. 1a (**a**) and the same set of hTERT-CRL-2097-derived cells as in Fig. 3a (**b**) were examined in western blot analysis for levels of total and phosphorylated STAT3, total and phosphorylated Akt, total and phosphorylated Erk1/2, and total and phosphorylated Src. Full-length blots/gels are presented in Supplementary Figure

We examined the activation status of three major factors downstream of EML4-ALK (i.e., STAT3, Akt and Erk1/2) by western blot (Fig. 4a and b). Mortal normal human fibroblasts CRL-2097/TR with and without EML4-ALK expression both showed increased levels of phosphorylated Akt when they became senescent, while the levels of phosphorylated Erk1/2 did not show a consistent change associated with EML4-ALK expression or increased PDL levels (Fig. 4a). Notably, a striking induction of STAT3 phosphorylation was observed in the EML4-ALK-expressing cells at PDL 4, which was decreased upon senescent proliferation arrest (PDL 6) but still at a higher level than that in the control cells at senescence (PDL 11) (Fig. 4a). Also in hTERT-transduced CRL-2097 fibroblasts, the expression of EML4-ALK resulted in remarkable induction of phosphorylated STAT3, while no increase in phosphorylated Akt and a slight increase in phosphorylated Erk1/2 were associated with EML4-ALK expression (Fig. 4b). These findings suggest that STAT3 functions as a downstream effector of EML4-ALK in both mortal and hTERT-transduced normal human fibroblasts, consistent with its involvement in both cellular senescence and transformation [36–39] and the previous studies directly linking EML4-ALK to STAT3 phosphorylation [40, 41]. MiR-21, a microRNA induced by STAT3 [42], was also upregulated by EML4-ALK in both mortal and hTERT-transduced fibroblasts (Supplementary Fig. S8). Although a decrease in phosphorylated Src was associated with cellular senescence in the presence or absence of EML4-ALK (Fig. 4a), the expression of EML4-ALK did not affect the phosphorylation level of Src (Fig. 4a and b), which is known to mediate acquired resistance to ALK TKIs [43].

### EML4-ALK regulates different signaling pathways in mortal and hTERT-transduced normal human fibroblasts

We performed RNA sequencing (RNA-seq) in duplicated samples of mortal CRL-2097/TR with and without EML4-ALK expression (Supplementary Fig. S9A; Supplementary Table S1) and hTERT-transduced CRL-2097 with and without EML4-ALK expression (Supplementary Fig. S9B; Supplementary Table S2). The analysis of the differentially expressed genes to KEGG (Kyoto Encyclopedia of Genes and Genomes) Pathways (https://www.genome.jp/kegg/) identified the cytokine-cytokine receptor interaction pathway as significantly upregulated by EML4-ALK expression in mortal CRL-2097/TR fibroblasts, along with some related and overlapping pathways involving tumor necrosis factor (TNF) or viral infection (Table 1 and Supplementary Table S3). The interferon (IFN)-α/β and IFN-γ signaling pathways, which have some genes in common with the above KEGG pathways, were also identified from Reactome Pathway Database (https://reactome.org/) as EML4-ALK-upregulated pathways in mortal CRL-2097/TR fibroblasts (Table 1 and Supplementary Table S3). Consistent with the EML4-ALK-induced phosphorylation of STAT3 (as above in Fig. 4), these cytokines and IFN signaling pathways include several STAT3-upregulated genes such as IL1B, CXCL8, SOCS3, IRF1 and IRF7 (Table 1 and Supplementary Table S3). The upregulation of IL1B and CXCL8 by EML4-ALK was confirmed by qRT-PCR (Supplementary Fig. S10). These data suggest that STAT3 may mediate the effect of EML4-ALK on activating the proinflammatory cytokine and IFN signaling cascades, which coordinately induce and maintain sustained DNA damage and senescent proliferative arrest in mortal normal human cells [39, 44]. Although cell cycle regulator genes are expected to change in expression upon senescence, EML4-ALK by itself in pre-senescent cells did not lead to significant enrichment of the pathways for cell cycle regulation (at https://david.ncifcrf.gov/: GO 0051726 regulation of cell cycle, FDR 0.90; GO 0007050 cell cycle arrest, FDR 0.90; GO 0007049 cell cycle, FDR 0.91). The signaling pathways involved in DNA damage response were also not significantly affected by EML4-ALK (GO 0006974 cellular response to DNA damage stimulus, FDR 0.99; GO 0006281 DNA repair, FDR 1.00), suggesting that EML4-ALK-induced DNA damage is not primarily due to impaired DNA repair.

**Table 1:** Pathway analysis of differentially expressed genes showing that EML4-ALK regulates different downstream pathways in mortal and immortalized human fibroblasts^a^

	Fold Enrichment	FDR^b^	Genes (Official Symbols)^c^							
**CRL-2097/TR: Upregulated by EML4-ALK**										
[KEGG Pathways]										
hsa05164: Influenza A	6.919	7.91E-05	SOCS3	OAS2	HSPA6	OAS3	DDX58	RSAD2	IFIH1	MX1
			IRF7	IL1B	CXCL10	TNFSF10	CXCL8	OAS1		
hsa04060: Cytokine-cytokine receptor interaction	5.662	8.95E-05	IL15RA	CCL8	TNFSF10	CCL3	CSF3	TNFSF13B	IL24	CXCL3
			CXCL5	CSF2	CXCL11	INHBA	IL1B	CXCL10	CXCL8	BMP2
hsa05162: Measles	6.465	2.18E-02	OAS2	HSPA6	OAS3	DDX58	MX1	IRF7	IFIH1	IL1B
			TNFSF10	OAS1						
hsa04668: TNF signaling pathway	7.233	3.18E-02	BIRC3	SOCS3	CXCL3	CSF2	IL1B	JUNB	CXCL10	MMP3
			MLKL							
hsa05168: Herpes simplex infection	5.169	4.71E-02	SOCS3	OAS2	IFIT1	OAS3	DDX58	IRF7	IFIH1	IL1B
			HLA-F	TAP1	OAS1					
[Reactome Pathways]										
R-HSA-909733: Interferon alpha/beta signaling	29.503	8.74E-23	MX2	OAS2	IFIT2	BST2	OAS3	ISG20	ISG15	GBP2
			IFI27	OASL	OAS1	IFIT3	IRF1	SOCS3	IFI6	IFIT1
			IFI35	RSAD2	IRF7	MX1	IFITM1	HLA-F		
R-HSA-877300: Interferon gamma signaling	14.295	1.19E-08	GBP5	OAS2	GBP7	OAS3	GBP4	GBP2	OASL	OAS1
			IRF1	SOCS3	IFI30	GBP1	IRF7	HLA-F		
**hTERT-CRL-2097: Upregulated by EML4-ALK**										
[KEGG Pathways]										
hsa04610: Complement and coagulation cascades	16.616	4.62E-05	PLAT	BDKRB2	PLAU	A2M	C1R	SERPING1	PROS1	CFH
			C1S							
**hTERT-CRL-2097: Downregulated by EML4-ALK**										
[KEGG Pathways]										
hsa04510: Focal adhesion	7.633	5.47E-02	ACTN4	ACTB	COL4A2	COL4A1	FLNA	MYL9	COL11A1	ITGA1
[Reactome Pathways]										
R-HSA-3000171: Non-integrin membrane-ECM interactions	25.781	4.11E-02	NTN4	COL4A2	HSPG2	COL4A1	COL11A1			

^a^The differentially expressed genes were analyzed to KEGG and Reactome Pathways Databases as described in Materials and methods

^b^False discovery rate

^c^Full names are listed in Supplementary Table S3

In hTERT-transduced CRL-2097 fibroblasts, instead of the above-mentioned cytokine and IFN signaling pathways, the complement and blood coagulation cascades signaling was identified as significantly upregulated by EML4-ALK expression (Table 1 and Supplementary Table S3). This pathway included A2M, PLAT and PLAU as STAT3-upregulated genes [45, 46] (the upregulation of these genes was confirmed by qRT-PCR, Supplementary Fig. S11), suggesting that STAT3 may also mediate the modulation by EML4-ALK of blood coagulation, which may have clinical implications in increased risk of disseminated intravenous coagulation in patients with EML4-ALK-positive cancer [47]. Consistently, a Japanese cohort also showed an upregulation of the blood coagulation pathway in EML4-ALK-positive lung cancer [24] (Supplementary Fig. S12 and Supplementary Table S4). We also found that some integrin and non-integrin components of focal adhesion and extracellular matrix (ECM) interactions were downregulated by EML4-ALK in hTERT-transduced CRL-2097 fibroblasts (Table 1 and Supplementary Table S3), which likely contributed to EML4-ALK-induced anchorage-independent growth via overcoming anoikis [48, 49].

### EML4-ALK also has in vitro transforming activity in hTERT-immortalized normal human bronchial epithelial cells but does not cause in vivo tumorigenicity

**Fig. 5:**
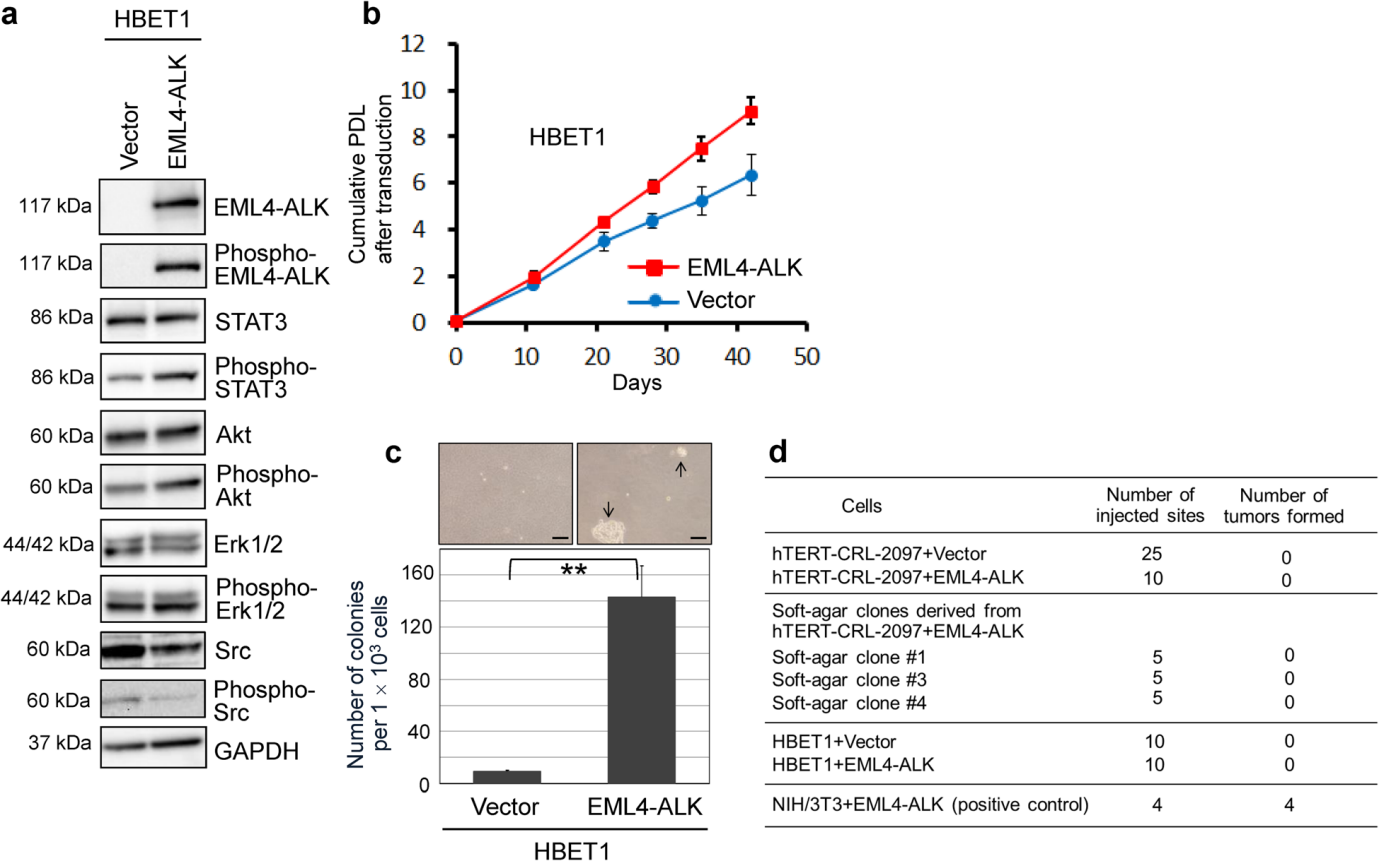
EML4-ALK promotes anchorage-independent growth in hTERT-immortalized normal human bronchial epithelial cells. **a** HBET1, an hTERT-immortalized normal human bronchial epithelial cell line, was transduced with the EML4-ALK lentiviral vector or the control vector as in Fig. 3. The cells at 28 days after transduction were examined in western blot analysis as in Figs. 3a and 4. Full-length blots/gels are presented in Supplementary Figure. **b** Cell proliferation curves of HBET1 expressing EML4-ALK or with the control vector. Cumulative PDL were calculated and plotted to days after transduction, as above. **c** Anchorage-independent growth of HBET1 cells expressing EML4-ALK. The HBET1 cells with EML4-ALK or the control vector were examined for anchorage-independent growth, as in Fig. 3d and S5C. Data analysis and presentation are also as in Fig. 3d and S5C. Arrows in a representative image indicate colonies that formed in soft-agar medium. Scale bars, 50 μm. ** *P* < 0.01. **d** Summary of tumorigenicity assay in NOD.SCID/Ncr mice. Cells were subcutaneously injected into each flank (5 × 10^6^ per flank) of mice at 6–10 weeks of age, followed by observation until 8 weeks after injection. In each mouse, one flank had EML4-ALK-expressing cells or soft-agar clones, and the other flank had cells with the control vector. Whereas NIH/3 T3 cells expressing EML4-ALK (2 × 10^6^ per flank, at both flanks of two mice) produced tumors *necessitating euthanasia*, no progressively growing tumors formed from any of hTERT-CRL-2097- and HBET1-derived cells

The transforming activity of EML4-ALK was also tested in hTERT-immortalized, normal human bronchial epithelial cells, which represent a cell type more relevant to NSCLC pathogenesis. For this purpose, we used a previously established cell line, HBET1, which has a tetraploid karyotype with no or few structurally abnormal chromosomes (Supplementary Fig. S13), maintains elongated telomeres and does not show anchorage-independent growth or in vivo tumorigenicity [14]. The HBET1 cells constitutively expressing EML4-ALK with its autophosphorylation (Fig. 5a) showed accelerated cell proliferation (Fig. 5b) and acquired anchorage-independent growth in soft agar (Fig. 5c), as observed above in hTERT-transduced fibroblasts. The increased levels of phosphorylation of STAT3 and Akt, but not of Erk1/2 or Src, were associated with EML4-ALK expression in HBET1 cells (Fig. 5a). Like in hTERT-transduced CRL-2097, PLAU and PLAT were upregulated by EML4-ALK in HBET1 cells as well (Supplementary Fig. S14).

To examine in vivo tumorigenicity, we injected HBET1 and hTERT-transduced CRL-2097 cells with and without EML4-ALK, along with the soft-agar clones derived from the EML4-ALK-expressing cells, subcutaneously into immunodeficient NOD.SCID/Ncr mice (Fig. 5d). None of these cells were able to form a growing tumor, while EML4-ALK-expressing mouse NIH/3 T3 cells as a positive control [4] consistently formed progressively growing tumors (Fig. 5d). These results suggest that the expression of EML4-ALK alone is not sufficient for hTERT-transduced normal human cells to acquire in vivo tumorigenicity under our experimental conditions.

## Discussion

This study for the first time performed the functional assays of an ALK fusion protein using normal human cells, which were mortal or hTERT-transduced. The major findings include: i) EML4-ALK, through its constitutive ALK kinase activity, induces mortal cells to undergo early senescence (Figs. 1 and 2); ii) EML4-ALK promotes hTERT-transduced cells to proliferate anchorage-independently (Figs. 3 and 5); iii) Accumulation of DNA damage in mortal but not hTERT-transduced cells (Fig. 1e and 3c) is associated with senescence induction; and iv) EML4-ALK regulates the different signaling pathways in mortal and hTERT-transduced cells (Table 1).

In mortal normal human fibroblasts, the expression of EML4-ALK caused senescent proliferation arrest earlier than natural replicative senescence, but later than senescence induced by oncogenic Ras (Fig. 1b and Supplementary Fig. S1), which was previously reported to cause severer DNA damage [12, 50]. The EML4-ALK expression is likely to cause DNA damage (indicated by γ-H2AX foci, Fig. 1e and f) through oncogene-induced DNA hyper-replication and DNA replication stress [51, 52], leading to the ATM-mediated p53 activation [28–30] (indicated by its serine 15 phosphorylation, Supplementary Fig. S2A) and then to upregulation of a p53-activated senescence inducer p21^WAF1^ [26] (Fig. 1d), as well as leading to DNA damage-associated upregulation of p16^INK4A^ [12, 53] (Fig. 1d). It is interesting to note a possible correlation between the severity of DNA damage and the degree of p16^INK4A^ upregulation in Ras-induced, EML4-ALK-induced and natural replicative senescence in this order (Fig. 1d).

A later phase of replicative lifespan of EML4-ALK-expressing mortal fibroblasts seemed to have selected against the cells with its higher expression level (compare Dox + at PDL 4 and 6 in Fig. 1a), and thus the cells with its lower expression level likely underwent cell proliferation prior to complete proliferation arrest. Interestingly, the kinase-dead mutant K589M showed a higher expression level than wild-type EML4-ALK in mortal fibroblasts (Fig. 2c) and hTERT-transduced fibroblasts continued to maintain high levels of wild-type EML4-ALK expression during expansion (Fig. 3a). These findings suggest that reduced expression of wild-type EML4-ALK and its phosphorylated form upon senescence of mortal fibroblasts most likely reflects its kinase-dependent senescence-inducing activity Nevertheless, we do not completely rule out a possibility that the vector-driven EML4-ALK expression might have been intrinsically mitigated during cell culture. Since our data suggest the accelerated accumulation of DNA damage as a major trigger for senescence induction, the high expression level of EML4-ALK itself may not necessarily be required for cellular senescence once enough DNA damage has been accumulated.

Our findings highlight a significant contrast of EML4-ALK activities between mortal and hTERT-transduced normal human cells. Since hTERT and telomerase are suggested to contribute to genome stability not only at telomeres but also at non-telomeric sites [54, 55], hTERT-transduced cells may become less vulnerable to telomeric and non-telomeric DNA damage, leading to a functional switch of EML4-ALK from senescence induction to in vitro transformation. Consistently, while the expression of EML4-ALK in mortal normal human fibroblasts activated the STAT3-mediated signaling to DNA damage and senescence involving proinflammatory cytokines and IFN pathways [39, 44] (Table 1 and Supplementary Table S3), these pathways were not activated in EML4-ALK-expressing hTERT-transduced fibroblasts, which instead showed changes in gene expression that could contribute to their anchorage-independent growth (Table 1 and Supplementary Table S3).

Chromosome abnormality, loss or mutation of *TP53*, or impairment of p16^INK4A^ response was not associated with EML4-ALK-induced anchorage-independent growth (Fig. 3e and Supplementary Fig. S7). These data are consistent with a previous finding that no or few chromosomal changes, other than an ALK-rearranging translocation, were observed in ALK fusion-positive tumors [1] and may reflect the molecular features of ALK fusion-positive tumors infrequently accompanying a genetic change affecting normal p53 and p16^INK4A^ functions [5]. Nonetheless, we speculate that other genetic or epigenetic events may also be involved in anchorage-independent growth induced by EML4-ALK. The higher efficiency of soft-agar colony formation in EML4-ALK-expressing HBET1 cells (Fig. 5c), compared with the counterparts of fibroblastic origin (Fig. 3d and Supplementary Fig. S6C), might be due to a genetic change(s) associated with numerical or structural chromosome aberrations that pre-existed in the original HBET1 cells (Supplementary Fig. S13) and/or due to hTERT immortalization-associated, pre-existing genetic and epigenetic changes frequently observed in epithelial cells [56] but not in fibroblasts [57].

Despite their ability of anchorage-independent growth, the EML4-ALK-expressing, hTERT-transduced normal human cells of fibroblastic origin (hTERT-CRL-2097) and of bronchial epithelial origin (HBET1) did not form tumors in immunodeficient mice under our experimental conditions (Fig. 5d), while mouse NIH/3 T3 cells expressing EML4-ALK were highly tumorigenic [4]. This contrast further highlights the importance of this study using normal human, and strongly suggests that additional genetic and/or epigenetic changes are required for development of EML4-ALK-positive tumors, prompting us to identify a factor(s) or event(s) that cooperates with EML4-ALK for acquisition of fully malignant phenotypes. Among candidate co-operating events to be examined is the inactivation of SETD2, a histone methyltransferase recently reported to be mutated in EML4-ALK fusion-driven lung adenocarcinoma [58].

## Conclusions

Our data indicate that the expression of hTERT is critical for EML4-ALK to manifest its in vitro transforming activity in human cells. Out data also suggest that STAT3 is a downstream factor of EML4-ALK and the different signaling pathways contributed to the different phenotypic outcomes in normal and hTERT-transduced cells. In addition, this study has established the isogenic pairs of human cell lines with and without EML4-ALK expression. We expect that these isogenic cells will be a novel in vitro cell system for screening and testing new drug candidates and investigating drug resistance, with significant implications in translational and clinical medicine towards better treatment of ALK fusion-induced human malignancy.

## Supplementary Information

**Additional file 1.**

## Data Availability

All data generated and analyzed in this study are included in this published article and its supplementary information files. Raw data and materials are available from the corresponding author upon request. RNA sequencing data have been deposited in the Gene Expression Omnibus (GEO) database with the accession numbers, GSE165137.

## Abbreviations

ALK: Anaplastic lymphoma kinase
EML4: Echinoderm microtubule associated protein like 4
NSCLC: Non-small cell lung carcinoma
PDL: Population doubling levels
hTERT: Human telomerase reverse transcriptase
TKI(s): Tyrosine kinase inhibitor(s)
SA-β-gal: Senescence-associated β-galactosidase
IFN: Interferon
