# Supplementary material for: EML4-ALK induces cellular senescence in mortal normal human cells and promotes anchorage-independent growth in hTERT-transduced normal human cells
Source: BMC Cancer. 2021 Mar 24;21:310. doi: 10.1186/s12885-021-07905-6 (PMC7992817; doi:10.1186/s12885-021-07905-6)
Supplement: Supplementary file 1 — Additional file 1. [file 12885_2021_7905_MOESM1_ESM.pdf]

EML4-ALK induces cellular senescence in mortal normal human cells and promotes anchorage-independent growth in hTERT-transduced normal human cells

Akihiko Miyanaga[a,b], Masaru Matsumoto[a,b], Jessica A. Beck[a], Izumi Horikawa[a], Takahiro Oike[a], Hirokazu Okayama[a], Hiromi Tanaka[c], Sandra S. Burkett[d], Ana I. Robles[a], Mohammed Khan[a], Delphine Lissa[a], Masahiro Seike[b], Akihiko Gemma[b], Hiroyuki Mano[e], Curtis C. Harris[a],*

Supplementary Figures

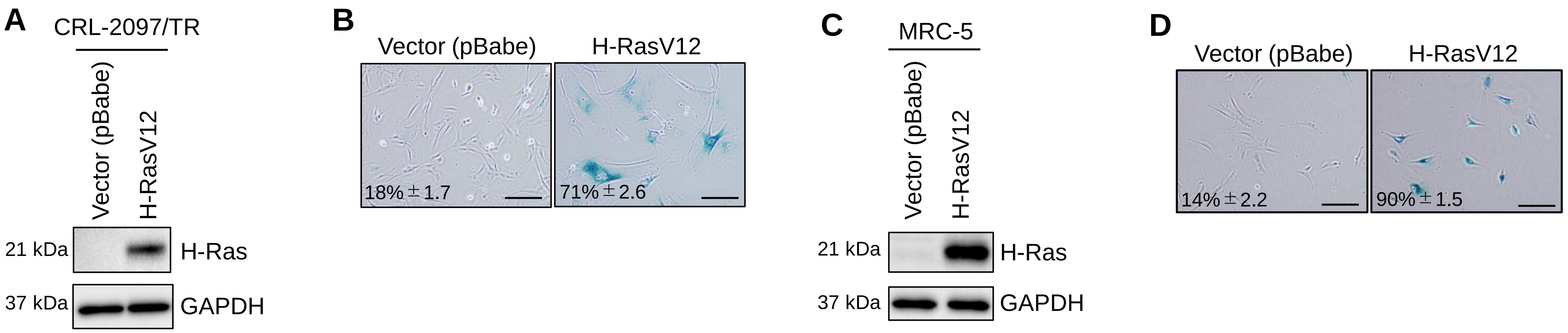

Supplementary Figure S1. Oncogenic Ras-induced cellular senescence in mortal normal human fibroblasts. CRL-2097/TR (A-B) and MRC-5 (C-D) fibroblasts were retrovirally transduced with oncogenic Ras (H-RasV12) and its control vector (pBabe). (A and C) Western blot analysis confirming the expression of H-RasV12. (B and D) Representative images of SA-β-gal staining with quantitative data of positive cells (mean ± s.d., as in Fig. 1c). Scale bars, 20 μm.

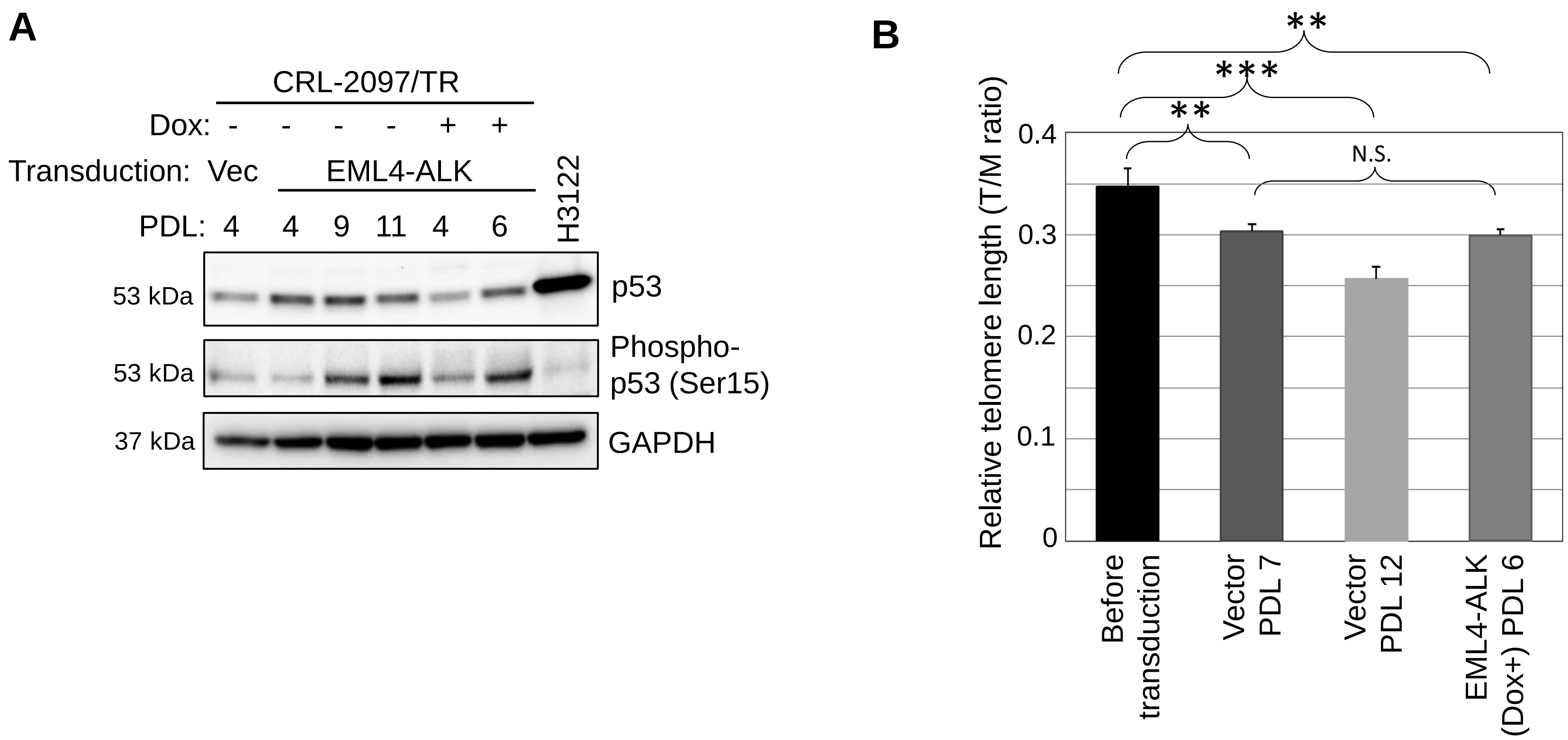

Supplementary Figure S2. DNA damage accumulated in EML4-ALK-expressing fibroblasts is partly attributed to telomere shortening. (A) Western blot analysis of p53. The same set of cells as in Fig. 1a were examined for levels of total p53 protein (top) and p53 phosphorylated at serine 15 (middle). (B) Quantitative analysis of telomere length by qPCR. CRL-2097/TR fibroblasts before transduction, those with control vector at PDL 7 (proliferating) and PDL 12 (senescent) and those with Dox-induced EML4-ALK expression at PDL 6 (senescent) were examined. Relative telomere lengths (T/M ratios) were determined as described in Materials and methods. Data (means ± s.d.) were from 5 independent experiments. ** $P < 0.01$; *** $P < 0.001$; N.S., not significant. Shown PDL is after transduction.

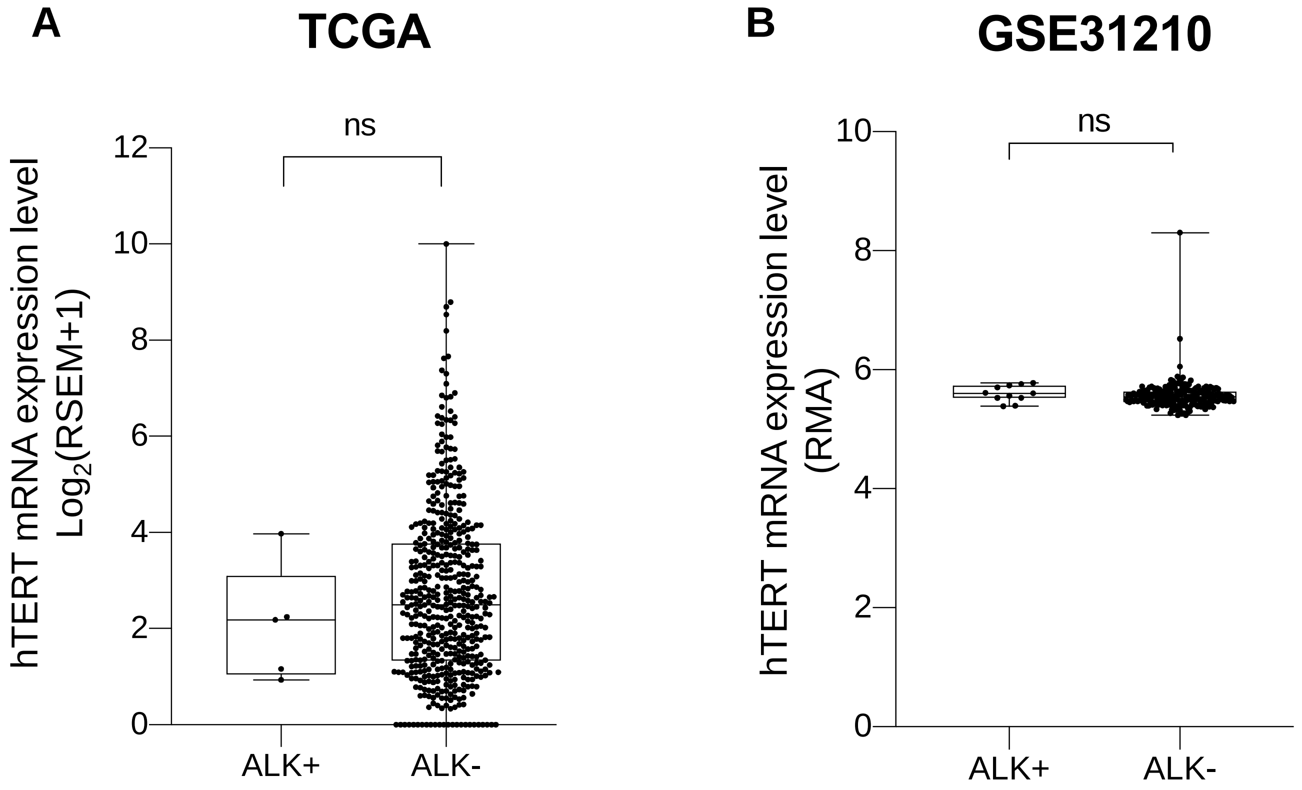

Supplementary Figure S3. Both ALK fusion-positive and -negative lung adenocarcinoma tissues express similar levels of hTERT. Transcript levels of hTERT in ALK fusion-positive and ALK fusion-negative lung adenocarcinomas were determined by RNA-seq (TCGA) (A) and microarrays (GSE31210) (B). Box plots show mRNA levels estimated by log_2-transformed RSEM (TCGA) or RMA (GSE31210). Groups were compared by Mann-Whitney U-test and Krustal-Wallis test (TCGA: ALK+, n=5; ALK-, n=505; GSE31210: ALK+, n=11; ALK-, n=215). ns; not significant.

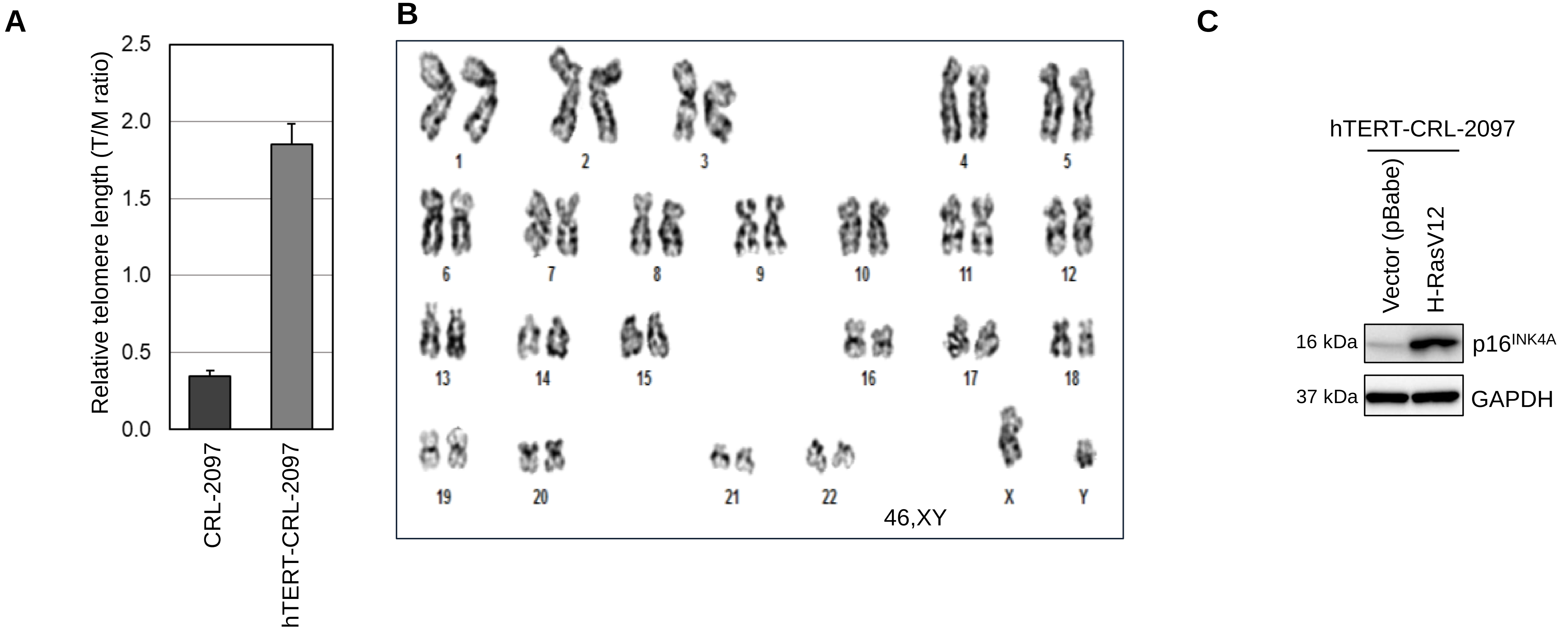

Supplementary Figure S4. Generation and characterization of hTERT-transduced CRL-2097 fibroblasts. To minimize a possibility of genetic alterations before, during and after hTERT transduction, early-passage well-proliferating CRL-2097 fibroblasts were transduced with hTERT on a 6-well, followed by mass culture expanding to a 10-cm plate without significant cell death under G418 selection, and then immediately used for EML4-ALK transduction in Fig. 3. The experiments in this supplementary figure used the cells after one or two more passages. (A) Elongated telomeres in hTERT-transduced CRL-2097 fibroblasts (hTERT-CRL-2097). Relative telomere lengths were analyzed as in Supplementary Fig. S2B and the result is shown in parallel to the original CRL-2097 fibroblasts. (B) A representative G-banding karyotype of hTERT-CRL-2097. All 20 metaphases analyzed (10 by G-banding and 10 by spectral karyotyping, SKY) had normal male karyotype, 46,XY. (C) Induction of $p16^{INK4A}$ in response to oncogenic Ras. hTERT-CRL-2097 cells were retrovirally transduced with H-RasV12 and its control vector (pBabe) and examined in western blot for $p16^{INK4A}$ expression. H-RasV12 induced senescence in a small fraction of cells (SA-β-gal positive 13.0 ± 1.6%, n=3), while EML4-ALK induced no sign of senescence in this cell line (Fig. 3b and 3c), possibly due to severer DNA damage caused by oncogenic Ras (Ref. 50).

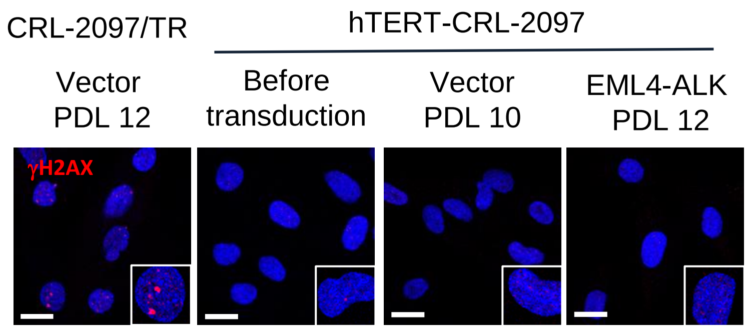

Supplementary Figure S5. Representative images of γ-H2AX immunofluorescence staining. The images merged with DAPI staining are shown for the cells in Fig. 3c. High-magnification images are in insets. Scale bars, 20 μm. Shown PDL is after transduction.

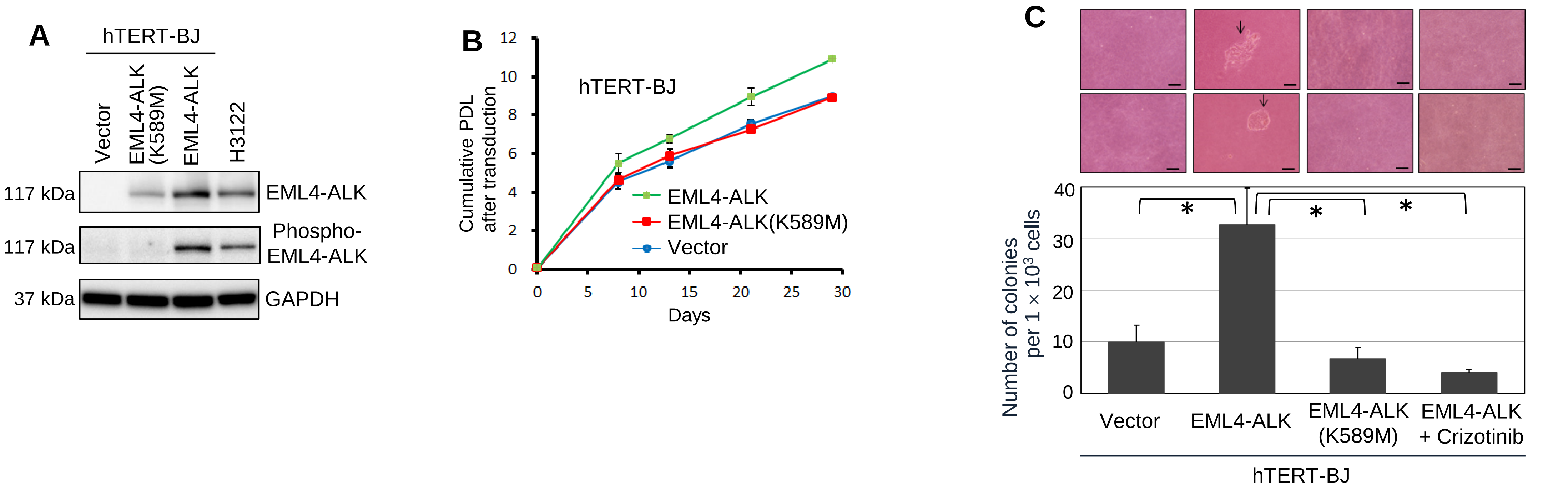

Supplementary Figure S6. EML4-ALK promotes anchorage-independent growth in hTERT-transduced normal human fibroblasts. (A) EML4-ALK expression in a second line of hTERT-transduced normal human fibroblasts, hTERT-BJ. The cells were transduced with the EML4-ALK and control vectors as in Fig. 3a, as well as with the EML4-ALK(K589M) vector with a kinase-dead mutation. The cells at 30 days after transduction, along with H3122, were examined in western blot analysis as in Fig. 1a and 3a. (B) Cell proliferation curves of hTERT-BJ expressing EML4-ALK or EML4-ALK(K589M) or with the control vector. Cumulative PDL were calculated and plotted to days after transduction, as above. (C) EML4-ALK-induced anchorage-independent growth of hTERT-BJ fibroblasts and its inhibition by Crizotinib or the kinase-dead mutation K589M. The hTERT-BJ cells expressing EML4-ALK, with and without 25 nM Crizotinib, those expressing EML4-ALK(K589M) and those with the control vector were examined for anchorage-independent colony formation in soft-agar medium, as in Fig. 3d. Data analysis and presentation are also as in Fig. 3d, with representative images in which arrows indicate colonies. Scale bars, 50 µm. * $P < 0.05$.

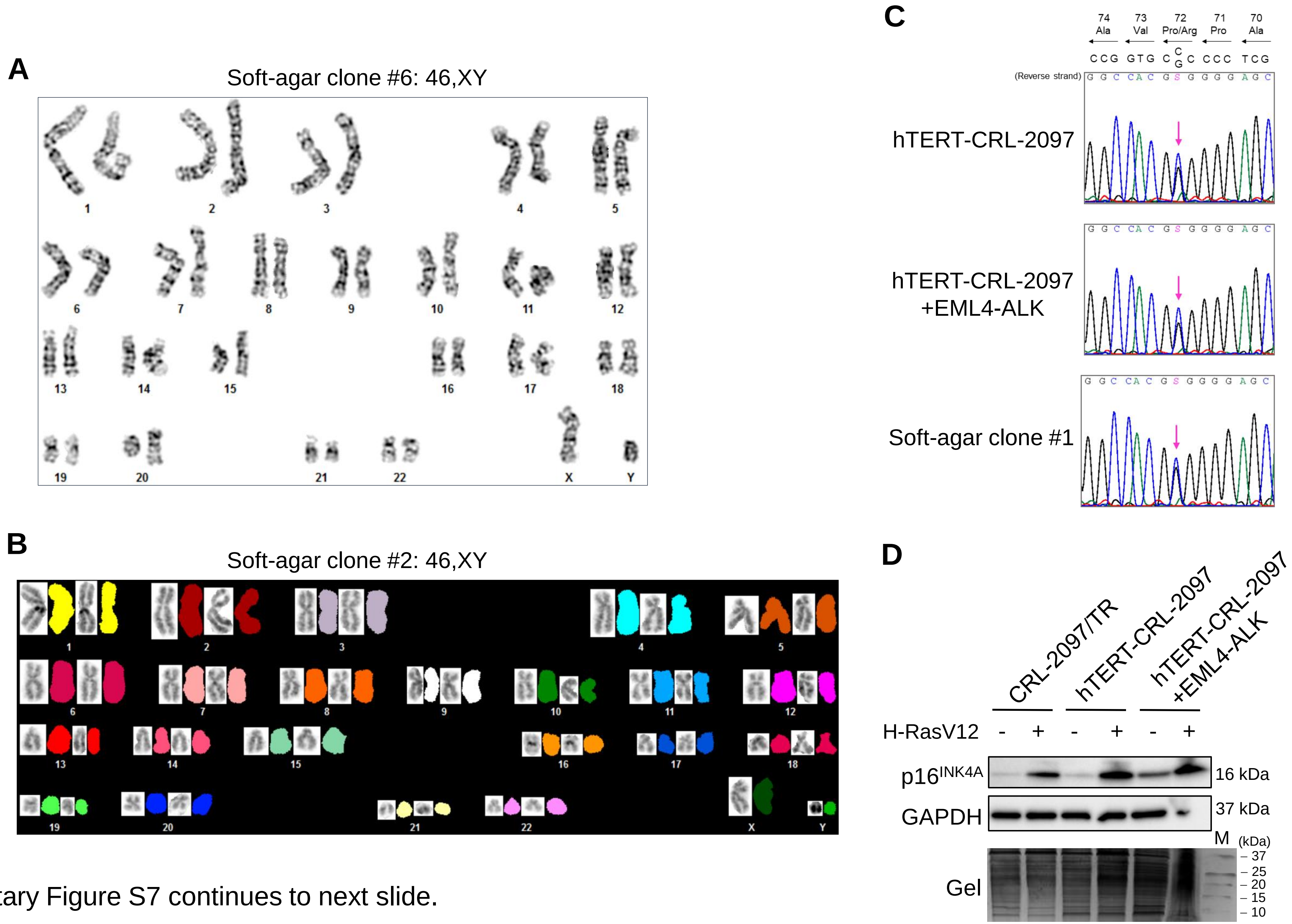

Supplementary Figure S7 continues to next slide.

Supplementary Figure S7. EML4-ALK does not cause chromosome aberrations, allelic loss of *TP53*, or impaired p16^{INK4A} response. (A) An example of G-banding analysis. Soft-agar clone #6 shows 46,XY. (B) An example of SKY analysis. Soft-agar clone #2 shows 46,XY. (C) Examples of Sanger sequencing of the RT-PCR products covering the entire coding sequence of TP53. The chromatograms around the polymorphic codon 72 (on reverse strand) are shown. The corresponding coding strand bases and amino acid residues are shown on the top. Red arrows indicate Pro/Arg heterozygosity at the codon 72. (D) Expression of p16^{INK4A} in response to oncogenic Ras. The retroviral transduction of H-RasV12 (+) and pBabe vector (-) were performed as in Supplementary Fig. S1 and S4C, followed by western blot analysis as in Fig. 1d and Supplementary Fig. S4C. A seemingly unusual GAPDH signal in the rightmost lane was likely due to distorted electrophoresis indicated by the stained gel (bottom), which serves as another loading control. M, size markers.

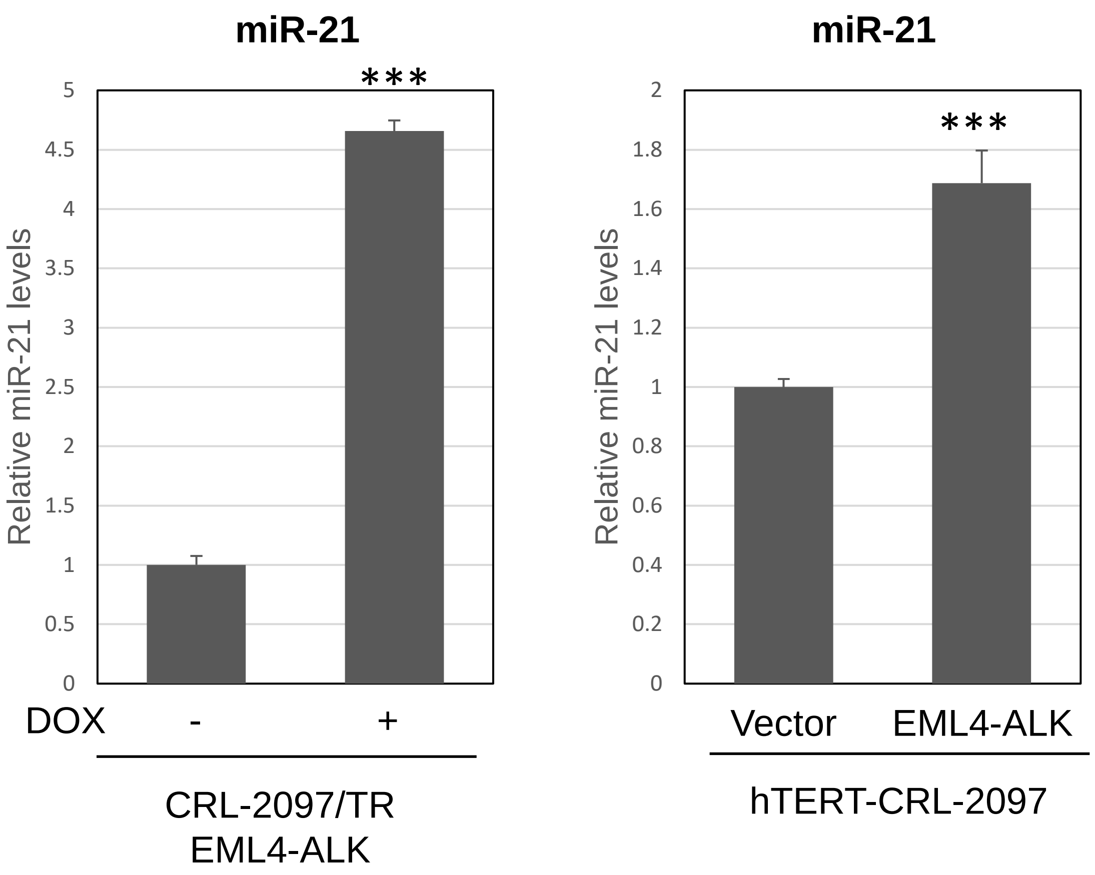

Supplementary Figure S8. EML4-ALK upregulates miR-21 in both mortal and hTERT-transduced human fibroblasts. Relative miR-21 levels are shown in mortal and hTERT-transduced fibroblasts with and without EML4-ALK expression. RNU48 was a control to normalize miR-21 levels. *** $P < 0.001$.

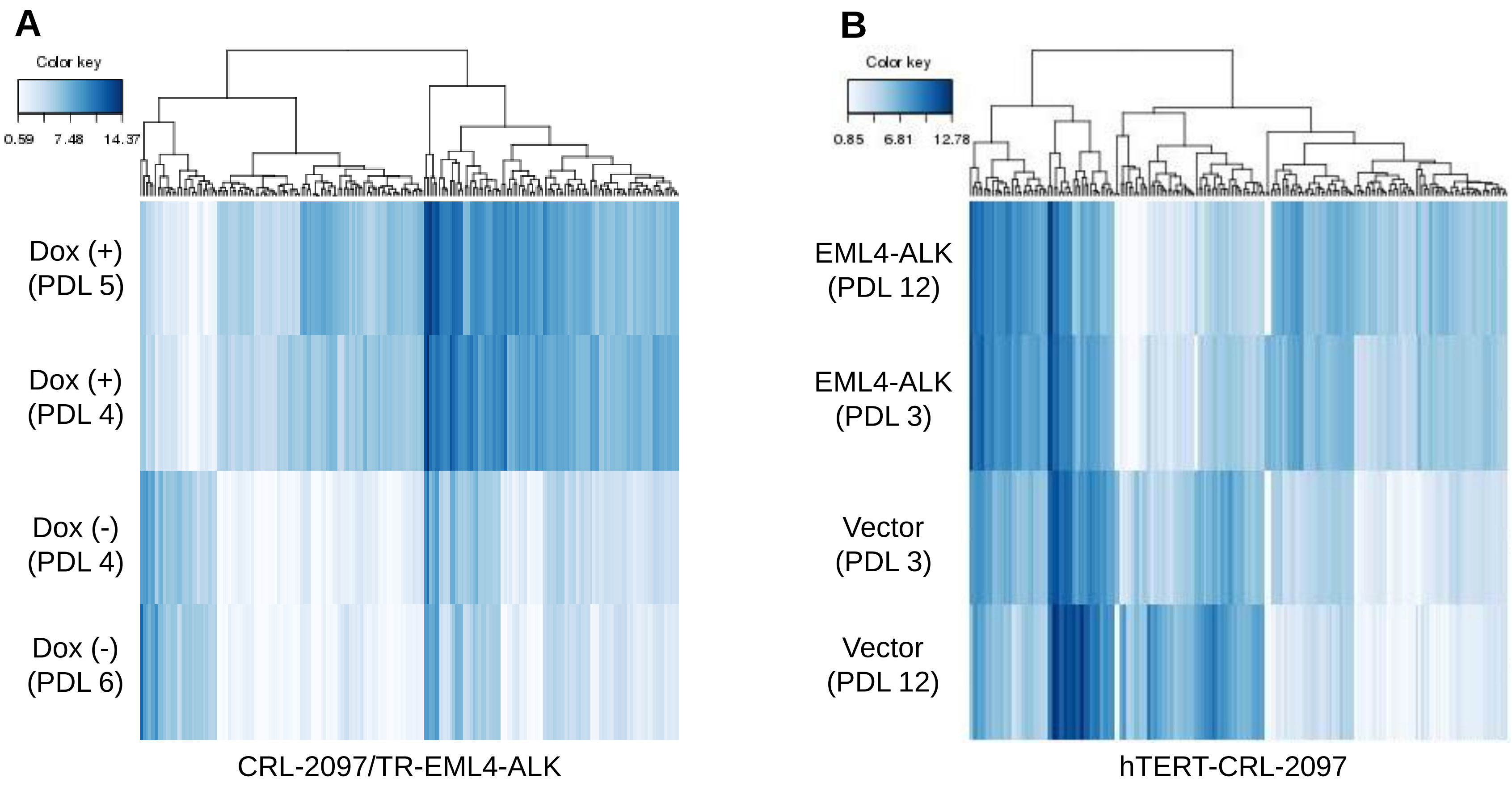

Supplementary Figure S9. Heatmaps of RNA-seq. (A) CRL-2097/TR with inducible EML4-ALK in the presence (+) or absence (-) of doxycycline (Dox). Both samples were biologically duplicated at different PDL as indicated in parentheses. (B) hTERT-CRL-2097 constitutively expressing EML4-ALK or with control vector. Both samples were biologically duplicated at different PDL as indicated in parentheses. Shown PDL is after transduction. Full data is available at GSE165137. Each data correspond as follows; GSM5026864: CRL-2097/TR-EML4-ALK Dox (+) (PDL 4), GSM5026865: CRL-2097/TR-EML4-ALK Dox (-) (PDL 6), GSM5026866: CRL-2097/TR-EML4-ALK Dox (+) (PDL 5), GSM5026867: CRL-2097/TR-EML4-ALK Dox (-) (PDL 4), GSM5026868: hTERT-CRL-2097-EML4-ALK (PDL 3), GSM5026869: hTERT-CRL-2097-Vector (PDL 3), GSM5026870: hTERT-CRL-2097-EML4-ALK (PDL 12), GSM5026871: hTERT-CRL-2097-Vector (PDL 12).

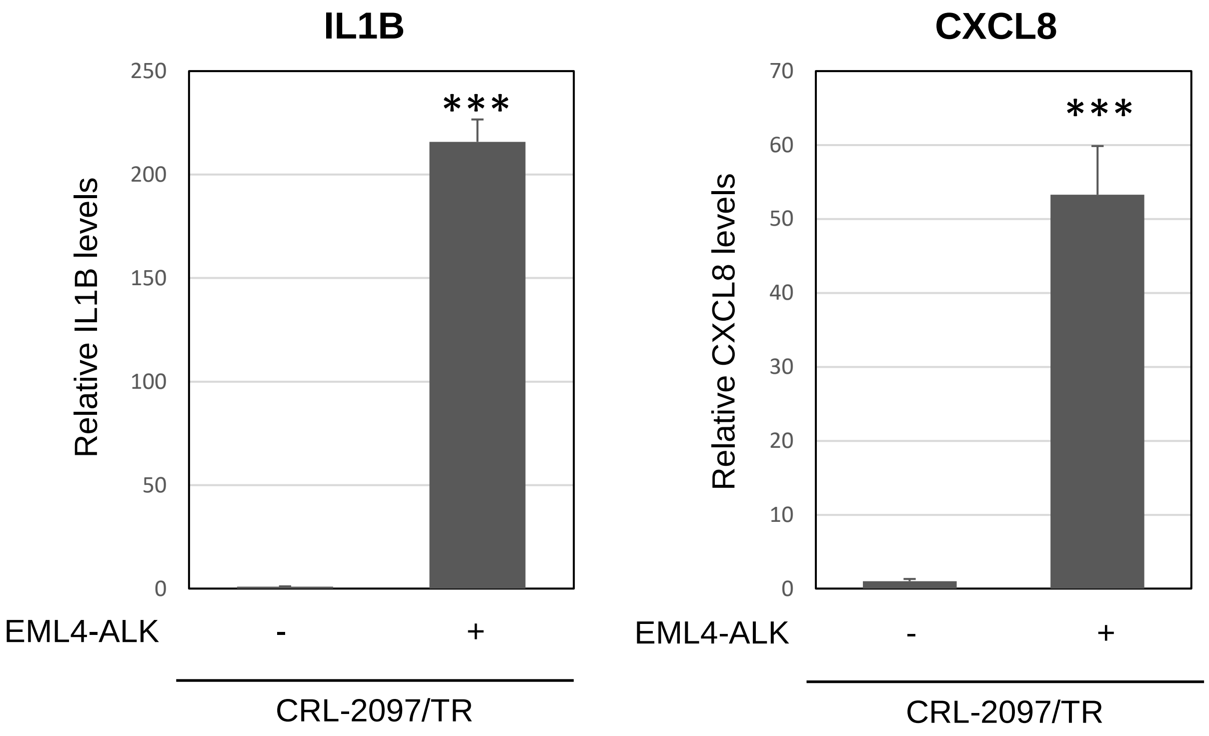

Supplementary Figure S10. EML4-ALK upregulates IL1B and CXCL8 in mortal human fibroblasts. Relative IL1B and CXCL8 levels are shown in CRL-2097/TR with and without EML4-ALK expression (PDL 5 and PDL 6, respectively). GAPDH was a control to normalize IL1B and CXCL8 levels. *** $P < 0.001$.

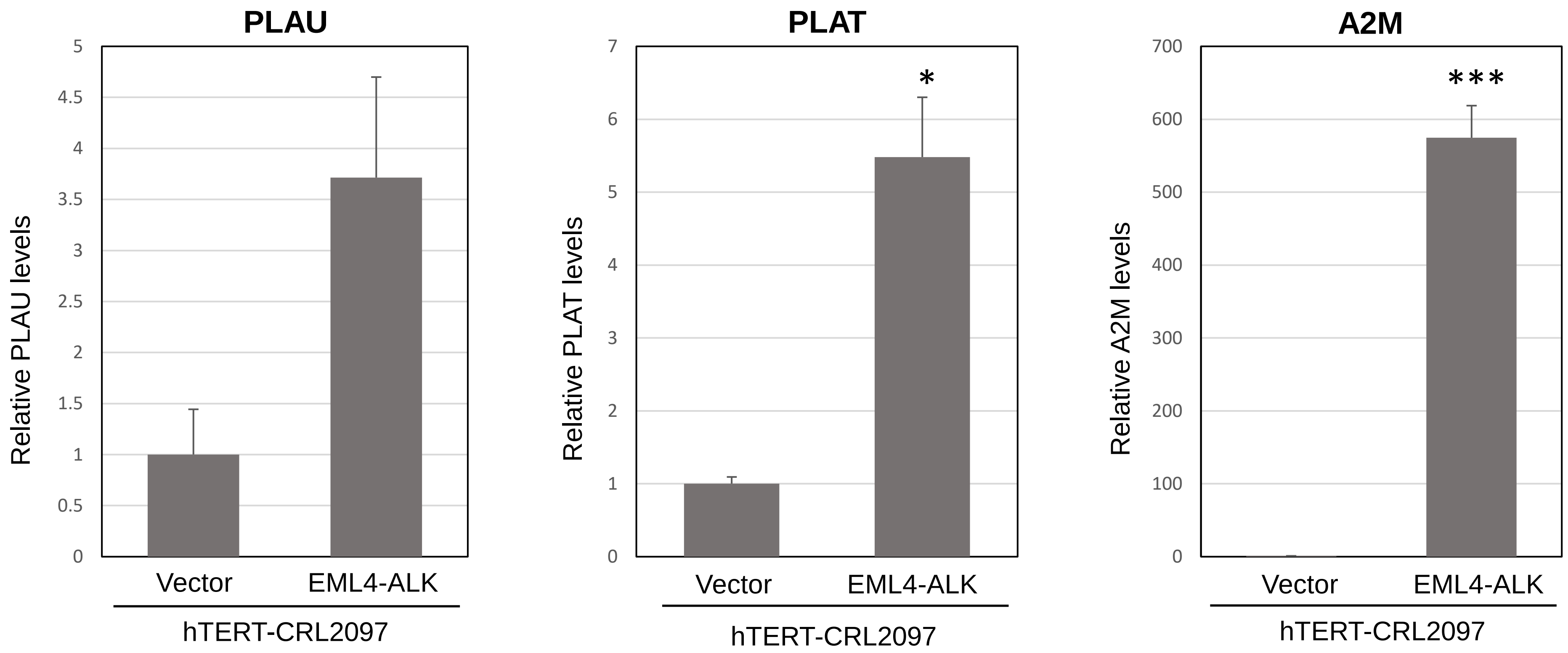

Supplementary Figure S11. EML4-ALK upregulates PLAU, PLAT and A2M in hTERT-transduced CRL-2097 with EML4-ALK. Relative PLAU, PLAT and A2M levels by Taqman qRT-PCR are shown in hTERT-transduced CRL-2097 with and without EML4-ALK expression. GAPDH was a control to normalize PLAU, PLAT and A2M levels. . * $P < 0.05$; *** $P < 0.001$.

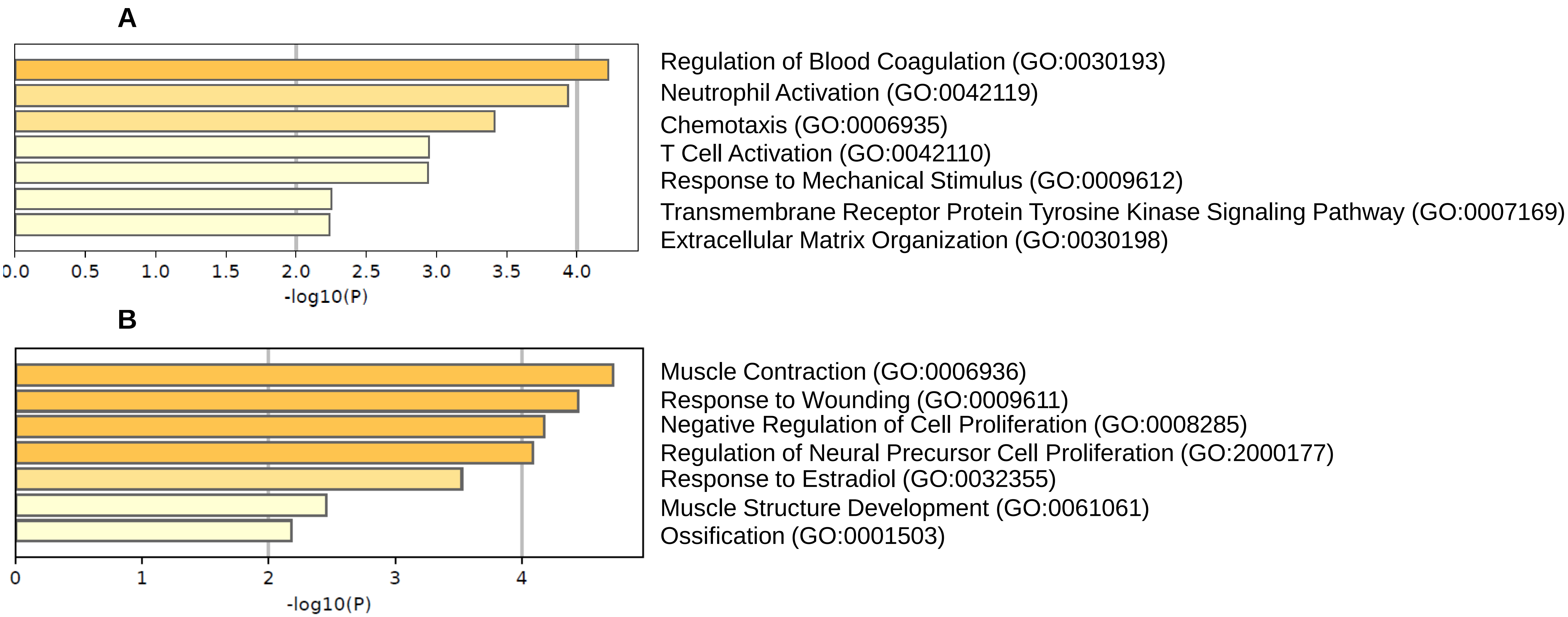

Supplementary Fig. S12. Upregulated (A) and downregulated (B) molecular functions commonly in ALK fusion-positive lung cancer tissues and EML4-ALK-expressing hTERT-CRL-2097 cells. The genes commonly upregulated or downregulated in ALK fusion-positive lung cancer tissues from Japanese cohort (compared to normal lung tissues) and EML4-ALK-expressing hTERT-CRL-2097 cells (compared to the vector control cells) were analyzed by Metascape (Zhou et al. Nat. Commun. 2019, 10:1523; http://metascape.org/gp/index.html#/main/step1). The whole list of the commonly regulated genes is available in Supplementary Table S4. The Gene Ontology Resource (GO) provides more details (http://geneontology.org/) on each pathway. "Log10(P)" is the p-value in log base 10.

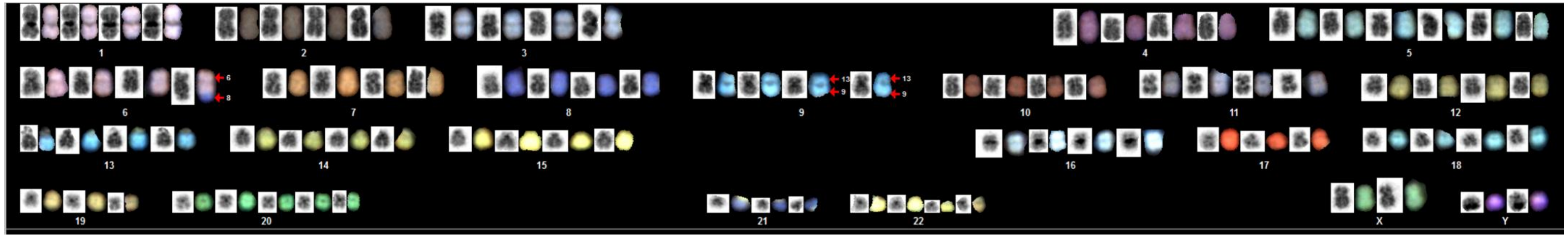

92,XXYY, +5,+5,-6,+t(6;8),-9,-9,+t(9;13)×2,-17,-19,+20,-21

Supplementary Figure S13. A representative SKY image of HBET1, an hTERT-immortalized normal human bronchial epithelial cell line. All 10 metaphases analyzed were tetraploid, 92,XXYY, with some numerical and a few structural abnormalities (red arrows).

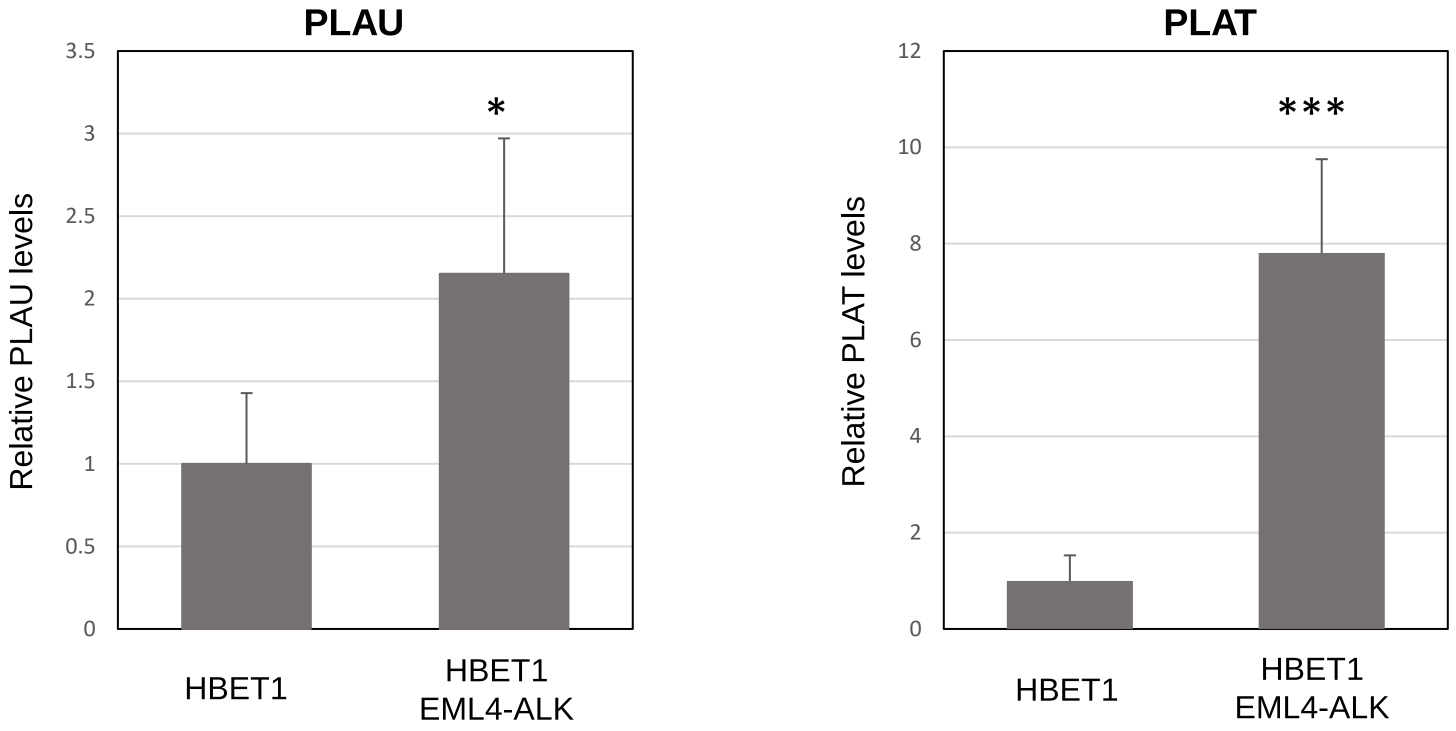

Supplementary Figure S14. EML4-ALK upregulates PLAU and PLAT in HBET1 with EML4-ALK. Relative PLAU and PLAT levels by Taqman qRT-PCR are shown in HBET1 cells with and without EML4-ALK expression. GAPDH was a control to normalize PLAU and PLAT levels. Unlike in hTERT-CRL-2097, A2M expression was below detection limit in this cell type. * $P < 0.05$; *** $P < 0.001$.

Supplementary Table S1. Differentially expressed genes in CRL-2097/TR

Ensembl	Symbol	log2 Fold Change	FDR
**[Upregulated by EML4-ALK]**			
ENSG00000131203.12	IDO1	11.00546151	8.43E-12
ENSG00000163220.10	S100A9	9.839903986	8.43E-12
ENSG00000206073.10	SERPINB4	9.621714237	8.43E-12
ENSG00000121858.10	TNFSF10	9.502665058	4.83E-10
ENSG00000280874.1	AC239868.2	9.218911373	3.09E-06
ENSG00000281134.1	AC239868.3	9.218911128	3.09E-06
ENSG00000200534.1	SNORA33	9.102514579	1.45E-06
ENSG00000134321.11	RSAD2	9.020993178	1.96E-10
ENSG00000143546.9	S100A8	8.922774251	2.83E-09
ENSG00000154451.14	GBP5	8.859958904	7.13E-08
ENSG00000169245.5	CXCL10	8.847259185	5.94E-09
ENSG00000277632.1	CCL3	8.79156421	1.23E-07
ENSG00000169248.12	CXCL11	8.76447017	7.13E-08
ENSG00000162654.8	GBP4	8.207154521	1.49E-08
ENSG00000057149.14	SERPINB3	8.180848347	9.13E-09
ENSG00000004468.12	CD38	7.776498366	3.94E-07
ENSG00000210825.1	SNORA40	7.679490113	2.56E-05
ENSG00000135114.12	OASL	7.592323241	2.56E-06
ENSG00000130303.12	BST2	7.549314721	7.58E-07
ENSG00000165949.12	IFI27	7.42238841	1.24E-07
ENSG00000225492.6	GBP1P1	7.311440013	6.11E-05
ENSG00000093134.13	VNN3	7.309482136	3.86E-07
ENSG00000108342.12	CSF3	7.271209708	6.77E-09
ENSG00000134326.11	CMPK2	7.221080137	1.22E-06
ENSG00000166920.10	C15orf48	7.148546052	2.57E-07
ENSG00000134339.8	SAA2	7.097235356	2.56E-06
ENSG00000079385.21	CEACAM1	6.933100331	7.99E-07
ENSG00000119922.8	IFIT2	6.809826952	2.62E-08
ENSG00000172183.14	ISG20	6.785976648	4.16E-07
ENSG00000185745.9	IFIT1	6.700399917	7.49E-10
ENSG00000108700.4	CCL8	6.686094847	1.50E-07
ENSG00000164400.5	CSF2	6.651623272	1.65E-07
ENSG00000119917.13	IFIT3	6.487041292	4.68E-09
ENSG00000213512.1	GBP7	6.473683606	0.0025814
ENSG00000173110.7	HSPA6	6.432567892	1.80E-05
ENSG00000089127.12	OAS1	6.380515544	5.37E-08
ENSG00000091137.11	SLC26A4	6.376438089	0.0003244
ENSG00000164266.10	SPINK1	6.331670449	0.0001139
ENSG00000175084.11	DES	6.320310211	1.61E-05
ENSG00000138646.8	HERC5	6.296149877	5.63E-06
ENSG00000157601.13	MX1	6.261083504	7.13E-08
ENSG00000168334.8	XIRP1	6.224679509	0.0032319
ENSG00000168421.12	RHOH	6.208870235	0.0076858

ENSG00000271216.1	LINC01050	6.157672474	0.0062683
ENSG00000196684.12	HSH2D	6.027858749	0.0056218
ENSG00000137745.11	MMP13	6.018760981	0.0003471
ENSG00000125538.11	IL1B	5.899562968	2.61E-07
ENSG00000129988.5	LBP	5.89816993	0.0006438
ENSG00000064886.13	CHI3L2	5.891602512	1.66E-05
ENSG00000050730.15	TNIP3	5.820902351	0.0007027
ENSG00000150551.10	LYPD1	5.802028226	8.78E-05
ENSG00000187608.8	ISG15	5.737431982	3.69E-05
ENSG00000266970.1	RP11-806H10.4	5.720161989	7.75E-06
ENSG00000166592.11	RRAD	5.691861969	5.63E-06
ENSG00000133321.10	RARRES3	5.63467886	9.62E-05
ENSG00000216490.3	IFI30	5.56365139	5.63E-06
ENSG00000163568.13	AIM2	5.415102608	4.47E-05
ENSG00000158859.9	ADAMTS4	5.32146625	0.0001645
ENSG00000232774.7	FLJ22447	5.316799703	0.0018738
ENSG00000170961.6	HAS2	5.245878977	2.55E-05
ENSG00000135604.9	STX11	5.242619003	0.0021796
ENSG00000162892.15	IL24	5.218961469	4.64E-06
ENSG00000197632.8	SERPINB2	5.142818623	0.0008159
ENSG00000170458.13	CD14	5.137248054	0.0002265
ENSG00000104951.15	IL4I1	5.070364218	5.41E-05
ENSG00000163735.6	CXCL5	5.018180853	0.0015773
ENSG00000143333.6	RGS16	5.005534738	0.0009152
ENSG00000130775.15	THEMIS2	4.97911383	2.00E-05
ENSG00000010030.13	ETV7	4.876667672	0.0004037
ENSG00000120217.13	CD274	4.79672041	9.62E-05
ENSG00000100342.20	APOL1	4.780449908	8.78E-05
ENSG00000181333.11	HEPHL1	4.769454938	0.0018429
ENSG00000168229.3	PTGDR	4.768806895	0.006639
ENSG00000168062.9	BATF2	4.760182918	3.72E-05
ENSG00000233705.6	SLC26A4-AS1	4.74932725	0.0044232
ENSG00000184557.4	SOCS3	4.745520662	2.41E-06
ENSG00000111012.9	CYP27B1	4.681215451	0.0008392
ENSG00000107201.9	DDX58	4.658076645	5.41E-05
ENSG00000164379.5	FOXQ1	4.643993691	0.0001139
ENSG00000169429.10	CXCL8	4.599650198	0.0001667
ENSG00000125845.6	BMP2	4.597972099	0.0003444
ENSG00000184979.9	USP18	4.57948308	3.72E-05
ENSG00000125144.13	MT1G	4.566655058	0.000699
ENSG00000102445.18	KIAA0226L	4.561347848	0.0007172
ENSG00000168961.16	LGALS9	4.5204297	0.0001331
ENSG00000183486.12	MX2	4.509380566	2.18E-05
ENSG00000101187.15	SLCO4A1	4.49624688	3.72E-05
ENSG00000151790.8	TDO2	4.488231764	0.0015626
ENSG00000111331.12	OAS3	4.479519001	6.11E-05
ENSG00000136689.18	IL1RN	4.473303045	0.0011233

ENSG00000122861.15	PLAU	4.470947208	7.48E-05
ENSG00000166670.9	MMP10	4.458430665	0.0002917
ENSG00000162551.13	ALPL	4.450458348	0.0047791
ENSG00000123610.4	TNFAIP6	4.447534113	0.0003331
ENSG00000111335.12	OAS2	4.396509658	0.0001139
ENSG00000023445.13	BIRC3	4.377707342	0.0099498
ENSG00000205364.3	MT1M	4.324247642	0.0032462
ENSG00000143924.18	EML4	4.296286217	0.0096868
ENSG00000178860.8	MSC	4.295301944	0.0091434
ENSG00000102524.11	TNFSF13B	4.287168707	0.0008392
ENSG00000163131.10	CTSS	4.283197089	0.0002025
ENSG00000074410.13	CA12	4.28163752	0.0006438
ENSG00000137959.15	IFI44L	4.262824661	0.001183
ENSG00000173432.10	SAA1	4.246969698	0.0003487
ENSG00000136514.2	RTP4	4.209066226	0.0047002
ENSG00000162772.16	ATF3	4.2082186	0.0001655
ENSG00000133106.14	EPSTI1	4.199694268	8.63E-05
ENSG00000169908.10	TM4SF1	4.137161435	7.48E-05
ENSG00000130066.16	SAT1	4.118358438	7.11E-05
ENSG00000115267.5	IFIH1	4.047103055	0.0002963
ENSG00000272841.1	RP3-428L16.2	4.044735537	0.0086212
ENSG00000028137.16	TNFRSF1B	4.027654719	0.0042213
ENSG00000100292.16	HMOX1	4.005386741	0.0018025
ENSG00000137496.17	IL18BP	3.99482688	0.0004482
ENSG00000135047.14	CTSL	3.985400466	7.11E-05
ENSG00000164283.12	ESM1	3.928239401	0.0007172
ENSG00000146278.10	PNRC1	3.881756866	0.0001012
ENSG00000134470.19	IL15RA	3.877451749	0.0003628
ENSG00000122641.9	INHBA	3.863676393	0.0050354
ENSG00000149968.11	MMP3	3.815761332	0.0041837
ENSG00000126709.14	IFI6	3.796857178	0.0018975
ENSG00000170525.18	PFKFB3	3.792366865	0.0091434
ENSG00000166396.12	SERPINB7	3.785477744	0.0006438
ENSG00000157214.13	STEAP2	3.763972838	0.0017134
ENSG00000068079.7	IFI35	3.73998332	0.0009058
ENSG00000025708.13	TYMP	3.698675923	0.0013867
ENSG00000162595.4	DIRAS3	3.681518958	0.001417
ENSG00000132196.13	HSD17B7	3.663507607	0.0014104
ENSG00000183691.4	NOG	3.649769107	0.0013735
ENSG00000204642.13	HLA-F	3.632944799	0.001936
ENSG00000117228.9	GBP1	3.625271731	0.0011233
ENSG00000185507.19	IRF7	3.576514688	0.0018747
ENSG00000130513.6	GDF15	3.530207902	0.0021796
ENSG00000151726.13	ACSL1	3.516487399	0.0028553
ENSG00000163734.4	CXCL3	3.478702348	0.0013925
ENSG00000185885.15	IFITM1	3.451598111	0.0047321
ENSG00000186480.12	INSIG1	3.446514331	0.0061024

ENSG00000137965.10	IFI44	3.400136996	0.0016347
ENSG00000135899.16	SP110	3.389710997	0.0013867
ENSG00000167772.11	ANGPTL4	3.359999043	0.0072576
ENSG00000125347.13	IRF1	3.354719468	0.0028553
ENSG00000162645.12	GBP2	3.332387268	0.0052487
ENSG00000141574.7	SECTM1	3.318993293	0.0029983
ENSG00000128335.13	APOL2	3.313141752	0.0019921
ENSG00000171223.5	JUNB	3.304823241	0.0025173
ENSG00000131459.12	GFPT2	3.257244281	0.002869
ENSG00000136630.12	HLX	3.24877073	0.0042213
ENSG00000140105.17	WARS	3.220017005	0.005246
ENSG00000163874.8	ZC3H12A	3.188828168	0.0042213
ENSG00000164647.8	STEAP1	3.185831401	0.004447
ENSG00000188313.12	PLSCR1	3.183955603	0.002877
ENSG00000240065.7	PSMB9	3.091411961	0.0093335
ENSG00000171621.13	SPSB1	3.078619141	0.0059943
ENSG00000133048.12	CHI3L1	3.069895916	0.0034
ENSG00000168394.10	TAP1	3.063395196	0.0053588
ENSG00000114268.11	PFKFB4	3.048890443	0.0095189
ENSG00000102802.9	MEDAG	3.025481997	0.0054597
ENSG00000157168.18	NRG1	2.937136765	0.0056367
ENSG00000168404.12	MLKL	2.936800322	0.0072639
ENSG00000123685.8	BATF3	2.934047337	0.0099498
ENSG00000198805.11	PNP	2.858168196	0.0069814
**[Downregulated by EML4-ALK]**			
ENSG00000270123.3	VTRNA2-1	-8.265050706	5.69E-07
ENSG00000202538.1	RNU4-2	-7.684021632	3.22E-05
ENSG00000207145.1	SNORA18	-7.490949231	2.99E-05
ENSG00000049540.16	ELN	-6.364338246	0.0003381
ENSG00000158270.11	COLEC12	-5.095344047	3.70E-05
ENSG00000102760.12	RGCC	-4.965763592	0.002015
ENSG00000146674.14	IGFBP3	-4.711135535	0.0009602
ENSG00000165092.12	ALDH1A1	-4.688144944	2.56E-05
ENSG00000198542.13	ITGBL1	-4.68327427	0.0045116
ENSG00000184347.14	SLIT3	-4.32177721	0.0016347
ENSG00000137463.4	MGARP	-4.158825505	0.0029421
ENSG00000181072.11	CHRM2	-4.151847738	0.0029179
ENSG00000186150.4	UBL4B	-4.069253786	0.002869
ENSG00000115457.9	IGFBP2	-4.003035051	0.0023527
ENSG00000103175.10	WFDC1	-3.972754301	0.0007563
ENSG00000206585.1	RNVU1-7	-3.747230951	0.0099498
ENSG00000104332.11	SFRP1	-3.698643343	0.0007511
ENSG00000022267.16	FHL1	-3.670211978	0.0005343
ENSG00000135069.13	PSAT1	-3.5092615	0.0007172
ENSG00000119938.8	PPP1R3C	-3.507768935	0.0008392
ENSG00000139211.6	AMIGO2	-3.488260929	0.006689

ENSG00000180914.10	OXTR	-3.430348445	0.0026783
ENSG00000138735.15	PDE5A	-3.399047685	0.0024645
ENSG00000163431.12	LMOD1	-3.329461301	0.002015
ENSG00000155760.2	FZD7	-3.292175351	0.0021259
ENSG00000113083.12	LOX	-3.078268353	0.0053824
ENSG00000107796.12	ACTA2	-3.00469875	0.0042213

Supplementary Table S2. Differentially expressed genes in hTERT-CRL-2097

Ensembl	Symbol	log2 Fold Change	FDR
**[Upregulated by EML4-ALK]**			
ENSG00000281902.1	AC091053.2	7.793784078	0.006246453
ENSG00000278144.1	NEAT1	7.467219995	0.009768796
ENSG00000166426.7	CRABP1	7.283592409	0.000718916
ENSG00000133048.12	CHI3L1	7.239890005	5.92E-09
ENSG00000171094.15	ALK	6.525621859	3.27E-12
ENSG00000122378.13	FAM213A	5.626420101	9.30E-07
ENSG00000189058.8	APOD	5.312978325	2.01E-14
ENSG00000115457.9	IGFBP2	5.225960617	2.72E-13
ENSG00000124875.9	CXCL6	5.195806911	0.002460041
ENSG00000102760.12	RGCC	5.153299196	9.30E-07
ENSG00000159167.11	STC1	5.083350433	1.46E-19
ENSG00000046653.14	GPM6B	4.953967637	0.000331503
ENSG00000103710.10	RASL12	4.924829004	0.005136512
ENSG00000141576.14	RNF157	4.819444784	0.000468872
ENSG00000225783.6	MIAT	4.812347217	5.27E-10
ENSG00000175899.14	A2M	4.72051183	1.02E-05
ENSG00000134247.9	PTGFRN	4.647445754	0.001735053
ENSG00000249992.1	TMEM158	4.597159106	2.53E-17
ENSG00000042062.11	FAM65C	4.36210296	1.88E-09
ENSG00000196611.4	MMP1	4.333131412	2.09E-09
ENSG00000124749.16	COL21A1	4.325559716	0.001123799
ENSG00000019991.15	HGF	4.202021544	2.42E-14
ENSG00000158270.11	COLEC12	4.028717768	1.11E-08
ENSG00000146072.6	TNFRSF21	3.974655496	0.000331503
ENSG00000108950.11	FAM20A	3.96878238	7.38E-14
ENSG00000151692.14	RNF144A	3.908911437	0.006021379
ENSG00000240801.1	AC132217.4	3.893009731	2.31E-06
ENSG00000170962.12	PDGFD	3.81218811	0.005368233
ENSG00000250722.5	SEPP1	3.785748778	0.008618972
ENSG00000172348.14	RCAN2	3.784175862	2.51E-09
ENSG00000258555.5	SPECC1L-ADORA2A	3.771833569	0.006021379
ENSG00000196549.10	MME	3.770616549	4.18E-18
ENSG00000088882.7	CPXM1	3.738256811	2.50E-09
ENSG00000151632.16	AKR1C2	3.635039837	0.000700058
ENSG00000187134.12	AKR1C1	3.57445286	8.30E-09
ENSG00000115594.11	IL1R1	3.510207976	4.80E-13
ENSG00000167244.18	IGF2	3.44561024	1.24E-06
ENSG00000128917.6	DLL4	3.442200991	0.006021379
ENSG00000164251.4	F2RL1	3.359962478	0.000355459
ENSG00000123496.7	IL13RA2	3.27246952	0.000700533
ENSG00000200320.1	SNORA63	3.241925093	0.001123799
ENSG00000138131.3	LOXL4	3.170770841	8.55E-09
ENSG00000117114.19	ADGRL2	3.152664171	0.008997017

ENSG00000109625.18	CPZ	3.144509082	4.31E-05
ENSG00000168916.15	ZNF608	3.007011796	0.00052274
ENSG00000183762.12	KREMEN1	3.003654553	4.98E-05
ENSG00000132386.10	SERPINF1	2.936364311	1.06E-07
ENSG00000196562.14	SULF2	2.887801844	7.80E-07
ENSG00000216775.2	RP1-152L7.5	2.832168095	1.58E-06
ENSG00000130702.13	LAMA5	2.74204469	0.005417138
ENSG00000162804.13	SNED1	2.74000426	9.68E-06
ENSG00000120885.19	CLU	2.716552266	0.00016812
ENSG00000177363.4	LRRN4CL	2.663566653	8.57E-05
ENSG00000158966.13	CACHD1	2.635182284	0.000409617
ENSG00000196460.12	RFX8	2.615958352	0.001565573
ENSG00000146966.12	DENND2A	2.611394477	0.003805553
ENSG00000135047.14	CTSL	2.541541052	7.12E-07
ENSG00000124882.3	EREG	2.524351625	8.32E-05
ENSG00000158747.13	NBL1	2.513578964	2.02E-05
ENSG00000178573.6	MAF	2.506120754	0.001533799
ENSG00000149256.14	TENM4	2.505597265	0.002569277
ENSG00000164647.8	STEAP1	2.495338999	8.49E-05
ENSG00000141574.7	SECTM1	2.48200794	0.009464717
ENSG00000198814.12	GK	2.462809545	0.000327695
ENSG00000116741.7	RGS2	2.458374858	0.000265532
ENSG00000169908.10	TM4SF1	2.438101594	5.31E-05
ENSG00000154065.16	ANKRD29	2.436159306	0.001123799
ENSG00000143248.12	RGS5	2.434380301	0.000331503
ENSG00000006468.13	ETV1	2.43434911	2.81E-05
ENSG00000143786.7	CNIH3	2.397893799	0.001123799
ENSG00000106976.19	DNM1	2.332432418	0.000109532
ENSG00000122861.15	PLAU	2.325865819	0.000127039
ENSG00000197635.9	DPP4	2.320762067	0.000882486
ENSG00000116729.13	WLS	2.310339556	1.72E-07
ENSG00000124785.8	NRN1	2.257451815	8.57E-05
ENSG00000137752.22	CASP1	2.248372201	3.36E-05
ENSG00000011465.16	DCN	2.244196481	3.83E-05
ENSG00000149131.15	SERPING1	2.236999825	1.02E-05
ENSG00000090776.5	EFNB1	2.225918601	0.007148107
ENSG00000173918.14	C1QTNF1	2.217336853	0.000910132
ENSG00000184500.14	PROS1	2.202167327	0.002051382
ENSG00000165633.12	VSTM4	2.194504544	0.00196393
ENSG00000115380.19	EFEMP1	2.148525849	2.31E-06
ENSG00000197632.8	SERPINB2	2.103446245	0.00076447
ENSG00000136859.9	ANGPTL2	2.077252506	4.41E-05
ENSG00000137767.13	SQRDL	1.998300988	5.15E-05
ENSG00000149090.11	PAMR1	1.994380439	0.008231357
ENSG00000134853.11	PDGFRA	1.964858762	0.000732716
ENSG00000166825.13	ANPEP	1.958829289	0.002147424
ENSG00000175745.11	NR2F1	1.942181479	0.003995182

ENSG00000111885.6	MAN1A1	1.923103155	0.000737625
ENSG00000101955.14	SRPX	1.909384131	0.000195028
ENSG00000013297.10	CLDN11	1.893526597	0.008620341
ENSG00000117155.16	SSX2IP	1.881893509	0.001081024
ENSG00000136960.12	ENPP2	1.87549133	0.001533799
ENSG00000121898.12	CPXM2	1.868513276	0.002603344
ENSG00000184557.4	SOCS3	1.860373301	0.001694919
ENSG00000139289.13	PHLDA1	1.850339737	0.000310088
ENSG00000136379.11	ABHD17C	1.842932156	0.002603344
ENSG00000105835.11	NAMPT	1.816582937	0.001252388
ENSG00000139329.4	LUM	1.816074966	0.001310934
ENSG00000211448.11	DIO2	1.81068976	0.009147523
ENSG00000139567.12	ACVRL1	1.805662298	0.000310088
ENSG00000000971.15	CFH	1.78950273	0.002307529
ENSG00000111913.15	FAM65B	1.769648032	0.003537509
ENSG00000168398.6	BDKRB2	1.761506753	0.003474738
ENSG00000176907.4	C8orf4	1.76080323	0.003474738
ENSG00000101000.5	PROCR	1.745537526	0.008859127
ENSG00000092068.18	SLC7A8	1.700415812	0.003474738
ENSG00000196405.12	EVL	1.682851469	0.002343012
ENSG00000121068.13	TBX2	1.626915348	0.003215005
ENSG00000159403.15	C1R	1.617704635	0.000375309
ENSG00000182326.14	C1S	1.606859016	0.000494041
ENSG00000101236.16	RNF24	1.596935853	0.003474738
ENSG00000112096.16	SOD2	1.53881787	0.003833758
ENSG00000146242.8	TPBG	1.527979887	0.008139281
ENSG00000135821.16	GLUL	1.516615759	0.004210583
ENSG00000177706.8	FAM20C	1.489465022	0.004970062
ENSG00000104368.17	PLAT	1.488384221	0.004667545
ENSG00000143387.12	CTSK	1.482984515	0.00300584
ENSG00000091136.13	LAMB1	1.300911058	0.008913855

[Downregulated by EML4-ALK]

ENSG00000187653.11	TMSB4XP8	-8.27499373	9.11E-18
ENSG00000207165.1	SNORA70	-8.077087558	1.35E-15
ENSG00000159251.6	ACTC1	-7.827687769	6.44E-05
ENSG00000276528.1	HOTAIRM1	-7.43155911	0.005984054
ENSG00000221716.1	SNORA11	-7.172849908	0.009450453
ENSG00000281052.1	U91328.1	-6.799935164	1.02E-05
ENSG00000276443.1	PVT1	-6.047700294	0.003319182
ENSG00000141052.17	MYOCD	-5.397884172	0.005984054
ENSG00000148053.15	NTRK2	-5.304053427	0.002502995
ENSG00000267601.1	RP11-323N12.5	-4.849910055	1.64E-09
ENSG00000157766.15	ACAN	-4.659211819	0.008454387
ENSG00000101384.11	JAG1	-4.405543618	0.001694919
ENSG00000118523.5	CTGF	-4.363860108	1.76E-06
ENSG00000107796.12	ACTA2	-4.314869877	0.001456722

ENSG00000122641.9	INHBA	-4.281672792	0.005312638
ENSG00000133110.14	POSTN	-4.277502405	0.003705081
ENSG00000173391.8	OLR1	-4.264904216	5.31E-05
ENSG00000049540.16	ELN	-4.064747052	0.000104913
ENSG00000187498.14	COL4A1	-3.994608615	0.000448639
ENSG00000076706.14	MCAM	-3.974623929	0.000350972
ENSG00000152049.6	KCNE4	-3.942420991	0.003319182
ENSG00000115468.11	EFHD1	-3.873749193	0.001344541
ENSG00000149596.6	JPH2	-3.777055475	0.003319182
ENSG00000133169.5	BEX1	-3.644700845	4.62E-08
ENSG00000099953.9	MMP11	-3.624539592	0.001593078
ENSG00000213949.8	ITGA1	-3.562776208	4.17E-05
ENSG00000170558.8	CDH2	-3.435942667	1.03E-08
ENSG00000130176.7	CNN1	-3.412655402	0.000354753
ENSG00000213145.9	CRIP1	-3.27202485	3.90E-05
ENSG00000074527.11	NTN4	-3.268856546	8.61E-05
ENSG00000135269.17	TES	-3.162628274	0.004811324
ENSG00000187634.10	SAMD11	-3.081335397	9.30E-07
ENSG00000111907.20	TPD52L1	-3.076283348	0.001215988
ENSG00000154678.16	PDE1C	-3.062778976	5.24E-05
ENSG00000117318.8	ID3	-3.016791976	2.09E-09
ENSG00000134871.17	COL4A2	-2.985946821	0.001593078
ENSG00000278371.2	AL442127.1	-2.952623423	0.000185256
ENSG00000060718.19	COL11A1	-2.872510593	1.39E-05
ENSG00000138650.8	PCDH10	-2.843189879	2.10E-05
ENSG00000134259.3	NGF	-2.836845854	1.35E-05
ENSG00000180914.10	OXTR	-2.77191315	1.41E-05
ENSG00000104881.14	PPP1R13L	-2.682088621	0.003354018
ENSG00000165949.12	IFI27	-2.681280047	3.44E-05
ENSG00000172403.10	SYNPO2	-2.636965007	0.001160289
ENSG00000140416.19	TPM1	-2.620693622	2.61E-05
ENSG00000137573.13	SULF1	-2.593122736	0.002147424
ENSG00000149591.16	TAGLN	-2.534936582	5.30E-05
ENSG00000103257.8	SLC7A5	-2.380273662	0.006080512
ENSG00000170390.15	DCLK2	-2.294369996	0.004839711
ENSG00000103175.10	WFDC1	-2.281999333	0.00275388
ENSG00000206190.11	ATP10A	-2.256044536	0.002894793
ENSG00000099860.8	GADD45B	-2.241149768	0.001123799
ENSG00000072163.18	LIMS2	-2.225309929	0.005417138
ENSG00000150938.9	CRIM1	-2.215758372	1.90E-05
ENSG00000169432.14	SCN9A	-2.187092314	0.004328193
ENSG00000111057.10	KRT18	-2.17967242	5.31E-05
ENSG00000159176.13	CSRP1	-2.095802174	0.000439223
ENSG00000134013.15	LOXL2	-2.026954652	0.000765539
ENSG00000125968.8	ID1	-1.991468883	0.000209294
ENSG00000115738.9	ID2	-1.973883783	0.000412782
ENSG00000142798.17	HSPG2	-1.954268152	0.005726287

ENSG00000101335.9	MYL9	-1.856733252	0.001658468
ENSG00000142871.15	CYR61	-1.78149506	0.000239989
ENSG00000187800.13	PEAR1	-1.769096688	0.000597847
ENSG00000164442.9	CITED2	-1.736143813	0.00246287
ENSG00000120129.5	DUSP1	-1.735261643	0.005200821
ENSG00000165996.13	HACD1	-1.73451374	0.00275388
ENSG00000112186.11	CAP2	-1.72188376	0.002460041
ENSG00000092820.17	EZR	-1.701909847	0.001533799
ENSG00000151414.14	NEK7	-1.66084233	0.003354018
ENSG00000196924.14	FLNA	-1.633664754	0.001565573
ENSG00000119280.16	C1orf198	-1.62672148	0.008018105
ENSG00000122786.19	CALD1	-1.624992026	0.005390351
ENSG00000100345.20	MYH9	-1.592470148	0.006434673
ENSG00000139211.6	AMIGO2	-1.578935528	0.002203778
ENSG00000130402.11	ACTN4	-1.539338162	0.003474738
ENSG00000187608.8	ISG15	-1.495042226	0.003319182
ENSG00000106366.8	SERPINE1	-1.465436075	0.007565742
ENSG00000139793.18	MBNL2	-1.364360004	0.009235824
ENSG00000075624.13	ACTB	-1.262572287	0.007006912

Supplementary Table S3. List of genes included in the identified KEGG and Reactome pathways

Ensembl	Symbol	log2 Fold Change	Full Name
**hsa05164: Influenza A**			
ENSG00000184557	SOCS3	4.75	suppressor of cytokine signaling 3
ENSG00000111335	OAS2	4.4	2'-5'-oligoadenylate synthetase 2
ENSG00000173110	HSPA6	6.43	heat shock protein family A (Hsp70) member 6
ENSG00000111331	OAS3	4.48	2'-5'-oligoadenylate synthetase 3
ENSG00000107201	DDX58	4.66	DExD/H-box helicase 58
ENSG00000134321	RSAD2	9.02	radical S-adenosyl methionine domain containing 2
ENSG00000115267	IFIH1	4.05	interferon induced with helicase C domain 1
ENSG00000157601	MX1	6.26	MX dynamin like GTPase 1
ENSG00000185507	IRF7	3.58	interferon regulatory factor 7
ENSG00000125538	IL1B	5.9	interleukin 1 beta
ENSG00000169245	CXCL10	8.85	C-X-C motif chemokine ligand 10
ENSG00000121858	TNFSF10	9.5	TNF superfamily member 10
ENSG00000169429	CXCL8	4.6	C-X-C motif chemokine ligand 8
ENSG00000089127	OAS1	6.38	2'-5'-oligoadenylate synthetase 1
**hsa04060: Cytokine-cytokine receptor interaction**			
ENSG00000134470	IL15RA	3.88	interleukin 15 receptor subunit alpha
ENSG00000108700	CCL8	6.69	C-C motif chemokine ligand 8
ENSG00000121858	TNFSF10	9.5	TNF superfamily member 10
ENSG00000277632	CCL3	8.79	C-C motif chemokine ligand 3
ENSG00000108342	CSF3	7.27	colony stimulating factor 3
ENSG00000102524	TNFSF13B	4.29	TNF superfamily member 13b
ENSG00000162892	IL24	5.22	interleukin 24
ENSG00000163734	CXCL3	3.48	C-X-C motif chemokine ligand 3
ENSG00000163735	CXCL5	5.02	C-X-C motif chemokine ligand 5
ENSG00000164400	CSF2	6.65	colony stimulating factor 2
ENSG00000169248	CXCL11	8.76	C-X-C motif chemokine ligand 11
ENSG00000122641	INHBA	3.86	inhibin subunit beta A
ENSG00000125538	IL1B	5.9	interleukin 1 beta
ENSG00000169245	CXCL10	8.85	C-X-C motif chemokine ligand 10
ENSG00000169429	CXCL8	4.6	C-X-C motif chemokine ligand 8
ENSG00000125845	BMP2	4.6	bone morphogenetic protein 2
**hsa05162: Measles**			
ENSG00000111335	OAS2	4.4	2'-5'-oligoadenylate synthetase 2
ENSG00000173110	HSPA6	6.43	heat shock protein family A (Hsp70) member 6
ENSG00000111331	OAS3	4.48	2'-5'-oligoadenylate synthetase 3
ENSG00000107201	DDX58	4.66	DExD/H-box helicase 58
ENSG00000157601	MX1	6.26	MX dynamin like GTPase 1
ENSG00000185507	IRF7	3.58	interferon regulatory factor 7
ENSG00000115267	IFIH1	4.05	interferon induced with helicase C domain 1
ENSG00000125538	IL1B	5.9	interleukin 1 beta
ENSG00000121858	TNFSF10	9.5	TNF superfamily member 10
ENSG00000089127	OAS1	6.38	2'-5'-oligoadenylate synthetase 1
**hsa04668: TNF signaling pathway**			
ENSG00000023445	BIRC3	4.38	baculoviral IAP repeat containing 3
ENSG00000184557	SOCS3	4.75	suppressor of cytokine signaling 3
ENSG00000163734	CXCL3	3.48	C-X-C motif chemokine ligand 3
ENSG00000164400	CSF2	6.65	colony stimulating factor 2
ENSG00000125538	IL1B	5.9	interleukin 1 beta
ENSG00000171223	JUNB	3.3	JunB proto-oncogene, AP-1 transcription factor subunit
ENSG00000169245	CXCL10	8.85	C-X-C motif chemokine ligand 10
ENSG00000149968	MMP3	3.82	matrix metallopeptidase 3

ENSG00000168404	MLKL	2.94	mixed lineage kinase domain like pseudokinase
**hsa05168: Herpes simplex infection**			
ENSG00000184557	SOCS3	4.75	suppressor of cytokine signaling 3
ENSG00000111335	OAS2	4.4	2'-5'-oligoadenylate synthetase 2
ENSG00000185745	IFIT1	6.7	interferon induced protein with tetratricopeptide repeats 1
ENSG00000111331	OAS3	4.48	2'-5'-oligoadenylate synthetase 3
ENSG00000107201	DDX58	4.66	DExD/H-box helicase 58
ENSG00000185507	IRF7	3.58	interferon regulatory factor 7
ENSG00000115267	IFIH1	4.05	interferon induced with helicase C domain 1
ENSG00000125538	IL1B	5.9	interleukin 1 beta
ENSG00000204642	HLA-F	3.63	major histocompatibility complex, class I, F
ENSG00000168394	TAP1	3.06	transporter 1, ATP binding cassette subfamily B member
ENSG00000089127	OAS1	6.38	2'-5'-oligoadenylate synthetase 1
**R-HSA-909733: Interferon alpha/beta signaling**			
ENSG00000183486	MX2	4.51	MX dynamin like GTPase 2
ENSG00000111335	OAS2	4.4	2'-5'-oligoadenylate synthetase 2
ENSG00000119922	IFIT2	6.81	interferon induced protein with tetratricopeptide repeats 2
ENSG00000130303	BST2	7.55	bone marrow stromal cell antigen 2
ENSG00000111331	OAS3	4.48	2'-5'-oligoadenylate synthetase 3
ENSG00000172183	ISG20	6.79	interferon stimulated exonuclease gene 20
ENSG00000187608	ISG15	5.74	ISG15 ubiquitin-like modifier
ENSG00000162645	GBP2	3.33	guanylate binding protein 2
ENSG00000165949	IFI27	7.42	interferon alpha inducible protein 27
ENSG00000135114	OASL	7.59	2'-5'-oligoadenylate synthetase like
ENSG00000089127	OAS1	6.38	2'-5'-oligoadenylate synthetase 1
ENSG00000119917	IFIT3	6.49	interferon induced protein with tetratricopeptide repeats 3
ENSG00000125347	IRF1	3.35	interferon regulatory factor 1
ENSG00000184557	SOCS3	4.75	suppressor of cytokine signaling 3
ENSG00000126709	IFI6	3.8	interferon alpha inducible protein 6
ENSG00000185745	IFIT1	6.7	interferon induced protein with tetratricopeptide repeats 1
ENSG00000068079	IFI35	3.74	interferon induced protein 35
ENSG00000134321	RSAD2	9.02	radical S-adenosyl methionine domain containing 2
ENSG00000185507	IRF7	3.58	interferon regulatory factor 7
ENSG00000157601	MX1	6.26	MX dynamin like GTPase 1
ENSG00000185885	IFITM1	3.45	interferon induced transmembrane protein 1
ENSG00000204642	HLA-F	3.63	major histocompatibility complex, class I, F
**R-HSA-877300: Interferon gamma signaling**			
ENSG00000154451	GBP5	8.86	guanylate binding protein 5
ENSG00000111335	OAS2	4.4	2'-5'-oligoadenylate synthetase 2
ENSG00000213512	GBP7	6.47	guanylate binding protein 7
ENSG00000111331	OAS3	4.48	2'-5'-oligoadenylate synthetase 3
ENSG00000162654	GBP4	8.21	guanylate binding protein 4
ENSG00000162645	GBP2	3.33	guanylate binding protein 2
ENSG00000135114	OASL	7.59	2'-5'-oligoadenylate synthetase like
ENSG00000089127	OAS1	6.38	2'-5'-oligoadenylate synthetase 1
ENSG00000125347	IRF1	3.35	interferon regulatory factor 1
ENSG00000184557	SOCS3	4.75	suppressor of cytokine signaling 3
ENSG00000216490	IFI30	5.56	IFI30 lysosomal thiol reductase
ENSG00000117228	GBP1	3.63	guanylate binding protein 1
ENSG00000185507	IRF7	3.58	interferon regulatory factor 7
ENSG00000204642	HLA-F	3.63	major histocompatibility complex, class I, F
**hsa04610: Complement and coagulation cascades**			
ENSG00000104368	PLAT	1.49	plasminogen activator, tissue type
ENSG00000168398	BDKRB2	1.76	bradykinin receptor B2

ENSG00000122861	PLAU	2.33	plasminogen activator, urokinase
ENSG00000175899	A2M	4.72	alpha-2-macroglobulin
ENSG00000159403	C1R	1.62	complement C1r
ENSG00000149131	SERPING1	2.24	serpin family G member 1
ENSG00000184500	PROS1	2.2	protein S
ENSG00000000971	CFH	1.79	complement factor H
ENSG00000182326	C1S	1.61	complement C1s
**hsa04510: Focal adhesion**			
ENSG00000130402	ACTN4	-1.54	actinin alpha 4
ENSG00000075624	ACTB	-1.26	actin beta
ENSG00000134871	COL4A2	-2.99	collagen type IV alpha 2 chain
ENSG00000187498	COL4A1	-3.99	collagen type IV alpha 1 chain
ENSG00000196924	FLNA	-1.63	filamin A
ENSG00000101335	MYL9	-1.86	myosin light chain 9
ENSG00000060718	COL11A1	-2.87	collagen type XI alpha 1 chain
ENSG00000213949	ITGA1	-3.56	integrin subunit alpha 1
**R-HSA-3000171: Non-integrin membrane-ECM interactions**			
ENSG00000074527	NTN4	-3.27	netrin 4
ENSG00000134871	COL4A2	-2.99	collagen type IV alpha 2 chain
ENSG00000142798	HSPG2	-1.95	heparan sulfate proteoglycan 2
ENSG00000187498	COL4A1	-3.99	collagen type IV alpha 1 chain
ENSG00000060718	COL11A1	-2.87	collagen type XI alpha 1 chain

Supplementary Table S4. List of genes differentially expressed commonly in ALK fusion-positive lung cancer tissues and EML4-ALK-expressing hTERT-CRL-2097 cells

Ensembl	Symbol	log2 Fold Change	log2 Fold Change
**[Upregulated genes]**		Cancer tissue	hTERT-CRL-2097 EML4-ALK
ENSG00000133048	CHI3L1	4.722976735	7.239890005
ENSG00000171094	ALK	6.063995504	6.525621859
ENSG00000115457	IGFBP2	2.18082479	5.225960617
ENSG00000124875	CXCL6	1.464747565	5.195806911
ENSG00000134247	PTGFRN	1.366747257	4.647445754
ENSG00000196611	MMP1	3.518571571	4.333131412
ENSG00000146072	TNFRSF21	1.993542755	3.974655496
ENSG00000108950	FAM20A	1.632866481	3.96878238
ENSG00000164251	F2RL1	1.448430516	3.359962478
ENSG00000138131	LOXL4	2.511884626	3.170770841
ENSG00000149256	TENM4	1.954449823	2.505597265
ENSG00000164647	STEAP1	2.504897107	2.495338999
ENSG00000169908	TM4SF1	2.090499946	2.438101594
ENSG00000006468	ETV1	1.179601958	2.43434911
ENSG00000143786	CNIH3	2.304127398	2.397893799
ENSG00000122861	PLAU	2.653156688	2.325865819
ENSG00000197635	DPP4	1.930819382	2.320762067
ENSG00000166825	ANPEP	1.150302825	1.958829289
ENSG00000136379	ABHD17C	1.25474546	1.842932156
ENSG00000211448	DIO2	1.652315399	1.81068976
ENSG00000101236	RNF24	1.539716123	1.596935853
ENSG00000146242	TPBG	1.97362122	1.527979887
ENSG00000177706	FAM20C	1.246842404	1.489465022
ENSG00000104368	PLAT	2.023671345	1.488384221
**[Downregulated genes]**			
ENSG00000233429	HOTAIRM1	-1.190545363	-7.43155911
ENSG00000118523	CTGF	-1.343569159	-4.363860108
ENSG00000076706	MCAM	-1.643149046	-3.974623929
ENSG00000149596	JPH2	-1.169302901	-3.777055475
ENSG00000133169	BEX1	-2.455074575	-3.644700845
ENSG00000213949	ITGA1	-1.205215944	-3.562776208
ENSG00000170558	CDH2	-1.537744059	-3.435942667
ENSG00000130176	CNN1	-1.898961557	-3.412655402
ENSG00000135269	TES	-1.058521966	-3.162628274
ENSG00000154678	PDE1C	-2.144607552	-3.062778976
ENSG00000117318	ID3	-1.3773924	-3.016791976
ENSG00000138650	PCDH10	-1.601953717	-2.843189879
ENSG00000172403	SYNPO2	-2.405036818	-2.636965007
ENSG00000149591	TAGLN	-1.239468321	-2.636965007
ENSG00000170390	DCLK2	-1.565310342	-2.294369996
ENSG00000103175	WFDC1	-2.170675927	-2.281999333

ENSG00000099860	GADD45B	-1.281426195	-2.241149768
ENSG00000072163	LIMS2	-1.612307526	-2.225309929
ENSG00000115738	ID2	-1.754184972	-1.973883783
ENSG00000101335	MYL9	-1.897782213	-1.856733252
ENSG00000187800	PEAR1	-1.890504181	-1.769096688
ENSG00000120129	DUSP1	-1.61609821	-1.735261643
ENSG00000112186	CAP2	-1.18096695	-1.72188376
ENSG00000122786	CALD1	-1.201899093	-1.624992026
